# Development of a framework to structure decision-making in environmental and occupational health: A systematic review and Delphi study^☆^

**DOI:** 10.1016/j.envint.2024.109209

**Published:** 2024-12-20

**Authors:** Emily Senerth, Paul Whaley, Elie Akl, Brandy Beverly, Pablo Alonso-Coello, Ezza Jalil, Jayati Khattar, Nicole R. Palmer, Andrew Rooney, Holger J. Schünemann, Kristina A. Thayer, Katya Tsaioun, Rebecca L. Morgan

**Affiliations:** a Department of Epidemiology, George Washington University, Milken Institute School of Public Health, 950 New Hampshire Ave NW #2, Washington, DC 20052, USA; b Evidence-based Toxicology Collaboration at Johns Hopkins Bloomberg School of Public Health, 615 North Wolfe Street, Rm: W7032, Baltimore, MD 21205, USA; c Lancaster Environment Centre, Lancaster University, Lancaster LA1 4YQ, UK; d American University of Beirut, P.O. Box 11-0236, Riad El-Solh, Beirut 1107 2020, Lebanon; e Division of the National Toxicology Program, National Institute of Environmental Health Sciences, National Institutes of Health, Department of Health and Human Services, P.O. Box 12233, Mail Drop K2-02, Research Triangle Park, NC, USA 27709; f Institut de Recerca Sant Pau (IR Sant Pau-CIBERESP), Sant Quintí 77-79 08041, Barcelona, Spain; g Centro Cochrane Iberoamericano, Sant Antoni Maria Claret, 167 08025, Barcelona, Spain; h Centro de Investigacion Biomédica en Red de Epidemiología y Salud Pública (CIBERESP), Madrid, Spain; i Cochrane Canada and McMaster GRADE Centres & Department of Health Research Methods, Evidence and Impact, McMaster University, Health Sciences Centre, Room 2C14, 1280 Main Street West, Hamilton, ON L8S 4K1, Canada; j Center for Public Health and Environmental Assessment, Chemical & Pollutant Assessment Division, Office of Research and Development, US Environmental Protection Agency, Building B (Room 211i), Research Triangle Park, NC, USA 27711; k School of Medicine, Case Western Reserve University, 10900 Euclid Ave, Cleveland, OH 44106, USA

**Keywords:** Systematic review, Delphi, GRADE, Decision-making, Environmental health, Occupational health, Decision framework

## Abstract

**Background:**

Environmental and occupational health (EOH) assessments increasingly utilize systematic review methods and structured frameworks for evaluating evidence about the human health effects of exposures. However, there is no prevailing approach for how to integrate this evidence into decisions or recommendations. Grading of Recommendations Assessment, Development and Evaluation (GRADE) evidence-to-decision (EtD) frameworks provide a structure to support standardized and transparent consideration of relevant criteria to inform health decisions. This study identifies and synthesizes available EOH decision frameworks and evaluates the applicability and usability of an existing GRADE EtD perspective to advance the development of a tailored EOH EtD framework.

**Methods:**

We performed a systematic review of MEDLINE, EMBASE, and Cochrane Library, and a manual search of gray literature to identify frameworks that inform decision-making about EOH exposures from the years 2011 to 2021. We abstracted and analyzed decision considerations from each framework through narrative synthesis. Next, we conducted a two-round Delphi process, engaging stakeholders from the following perspectives within environmental and occupational health: risk assessment and management, nutrition and food safety, cancer, and socio-economic analysis. Panelists rated the relevance and wording of each consideration on a 7-point Likert scale and provided free-text comments during both phases. Considerations that did not meet predetermined thresholds were excluded.

**Results:**

Out of 5,196 unique references, we identified 22 published reports of EOH decision frameworks. We identified another 16 frameworks in a search of gray literature, totaling 38 source frameworks. We abstracted 560 individual decision considerations from these frameworks, 104 of which may contribute additional information to the guidance, scope, context, or assessment criteria of the GRADE EtD framework. In round 1 of the Delphi study, 50 decision considerations were aggregated or removed, and 9 were aggregated or removed after round 2, for a final total of 47. No new decision considerations were added in either round. We identified several differences between decision criteria that are applied in EOH and the GRADE EtD framework, including vocabulary that is specific to EOH (e.g., toxicity, the precautionary principle), and granularity of the EOH decision considerations (e.g., detailed signaling questions to assess feasibility and resources required). However, this study did not identify any EOH decision criteria that are completely distinct from the GRADE EtD framework.

**Conclusions:**

Findings of this mixed-methods study comprise a foundation for a GRADE EtD that is applicable for use in EOH decision-making, with implications for approaches to regulation of environmental and occupational exposures and the formulation of recommendations for interventions to prevent or mitigate undesirable health and other consequences.

## 1. Introduction

According to a 2016 report from the World Health Organization (WHO), 24 % of global deaths are attributable to modifiable environmental risks. Healthier environments, including healthier workplace conditions, could prevent almost one quarter of the global burden of disease, or 13.7 million deaths per year. (Prüss-Üstün et al., 2016) Environmental health risks are assessed through a stepwise process involving hazard identification, dose–response assessment, exposure assessment, and risk characterization. (Council et al., 2009) This process yields evidence that may be used to inform regulation of exposures that are linked to undesirable health outcomes, or recommendations for interventions to prevent or mitigate risk. (Morgan et al., 2018).

The Grading of Recommendations Assessment, Development and Evaluation (GRADE) Working Group was established in 2000 as an international collaboration of methodologists, guideline developers, biostatisticians, clinicians, and public health experts. (Welcome to the GRADE working group: From evidence to recommendations – transparent and sensible [Internet], 2022) The Working Group has developed and continually improved an approach to assessing certainty in the evidence to answer questions about the effect of an intervention or exposure on human health outcomes. (Grading quality of evidence and strength of recommendations, 2004; Guyatt et al., 2008; Morgan et al., 2016) The GRADE approach to evidence appraisal has been adapted by the National Toxicology Program’s (NTP) Office of Health Assessment and Translation (OHAT), the Navigation Guide, and WHO, among other stakeholders, to support efforts to answer questions about the effects of environmental or occupational exposures. (Morgan et al., 2016; Woodruff and Sutton, 2014; National Toxicology Program, 2019; World Health Organization, 2014).

Certainty in the estimates of effects of exposures is one important consideration among several that should inform policymaking, regulation, priority-setting, or selection of one intervention over an alternative. After evidence has been collected and certainty has been assessed, the GRADE Evidence-to-Decision (EtD) frameworks support groups in making informed judgments about the pros and cons of different options, and provide a structure for recording and reporting these judgements, including reasons for disagreement. (Alonso-Coello et al., 2016) The GRADE EtD framework is comprised of twelve assessment criteria (priority of the problem, desirable effects, undesirable effects, values, balance of effects, certainty of evidence, resources required, certainty of resources required, cost effectiveness, equity, acceptability, and feasibility), which are informed by prompting questions (“detailed judgements”) that facilitate discussion and clarify what information used to inform the main criteria judgments. The GRADE EtD framework also includes scope and context criteria that are intended to help groups pre-specify the perspective, setting, and stakeholders that establish the conditions under which the decision will be made. The intended result is an evidence-informed recommendation or decision that is transparent and can be fully explained to stakeholders. (Gray and Cohen, 2012; Bero et al., 2020; Elliott and Resnik, n.d.; Hart, 2020; Grandjean and Ozonoff, 2013).

GRADE EtD frameworks have been tested and applied to five perspectives: individual clinical decisions, population-level clinical decisions, health system and public health decisions, coverage decisions, and tests. (Moberg et al., 2018; Morgan et al., 2018; Schünemann and Mustafa, 2017; Mustafa et al., 2017; Mustafa et al., 2017; Mustafa et al., 2017; Mustafa et al., 2017; Rehfuess et al., 2019; Neumann et al., 2016; Norris et al., 2021) However, the GRADE EtD framework is rarely used in the context of environmental and occupational health (EOH). (Morgan et al., 2016) As EOH decision-makers often rely on low certainty evidence to inform decisions that will impact broad stakeholder populations, we hypothesized that the GRADE EtD may facilitate systematic and transparent consideration of additional criteria with beneficial implications for the development and implementation of EOH recommendations and regulations. Examples of some common decisions in environmental and occupational health include selection of contaminated sites requiring remediation, selection of exposure thresholds for substances of concern, selection of an alternative to a known hazard, recommendations for remediation actions, or recommendations for interventions to mitigate harmful exposures. (US EPA, 2015; Asbestos Part 1; Chrysotile Asbestos; Regulation of Certain Conditions of Use Under the Toxic Substances Control Act (TSCA) [Internet], 2024; WHO global air quality guidelines: Particulate matter (PM2.5 and PM10), ozone, nitrogen dioxide, sulfur dioxide and carbon monoxide [Internet], 2021; Sands, 2017; Frisch, 2024; US EPA, 2015; Kiederer et al., 2024).

This project aims to further explore EOH decision-making by comparing existing EOH decision frameworks and the GRADE EtD to identify any gaps in content, structure, or terminology that present opportunities to improve the suitability of the GRADE EtD for use in EOH. We also aimed to elicit feedback from subject matter experts to evaluate the relevance of decision considerations that we identified through a systematic review, and to identify any considerations that were not evident in the literature. A subsequent manuscript will present the final EtD framework and provide guidance on its use.

## 2. Methods

### 2.1. Systematic review

#### 2.1.1. Protocol and search strategy

We conducted a systematic review of decision-making frameworks used in environmental and occupational health that updates and extends a prior review by Norris, et.al. on the of the same subject, followed by a narrative synthesis of the criteria and detailed judgments described in the included frameworks. (Morgan et al., 2018; Norris et al., 2021) Throughout, we adopted an inductive approach to identify considerations that are used to inform EOH decision-making, building on the deductive approach used to develop our foundational decision framework, the *GRADE EtD for health system and public health decisions*.

**Fig. 1.**
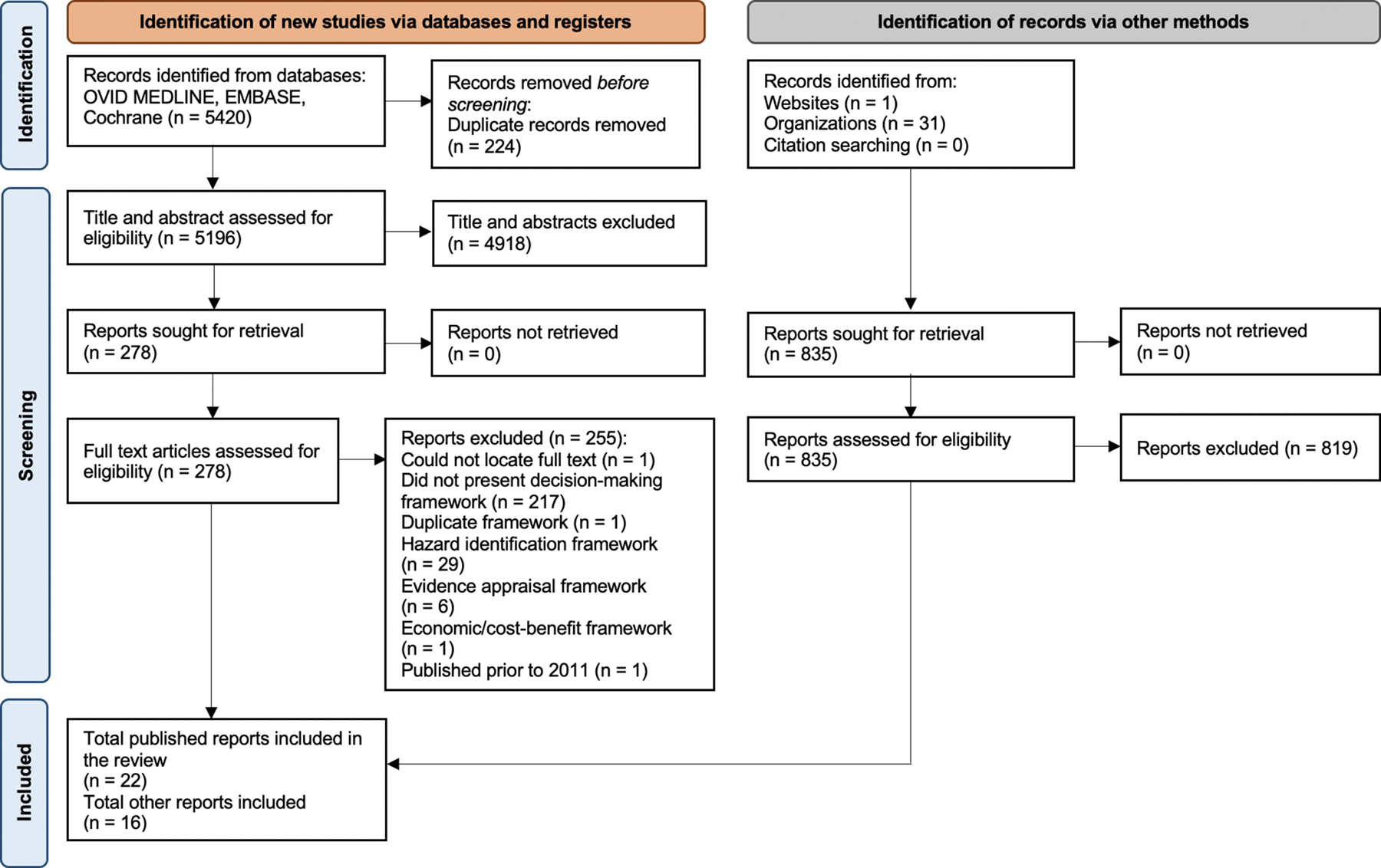
Preferred Reporting Items fo r Systematic Reviews and Meta-Analyses (PRISMA) 2020 flow diagram.

The systematic review protocol was registered on PROSPERO (CRD42022316686) and results are reported in the Preferred Reporting Items for Systematic Reviews and Meta-Analysis (PRISMA) format. (Aromataris and Munn, 2020; Page et al., 2021; Senerth et al., 2022) (Fig. 1) The search strategy was developed in consultation with an experienced health sciences librarian using a combination of free (keywords) and controlled (MeSH) vocabularies and translated into the following databases: MEDLINE via PubMed, EMBASE, and Cochrane Library. (Supplement A) The search was restricted to materials published in English within 10 years prior to the starting date of the review (September 26, 2021) to capture frameworks published both before and after publication of the GRADE EtD that are plausibly still in use. Additionally, we conducted a manual search of gray literature, including websites of government, professional, and public health organizations that produce health guidelines and the federal register. We used the Himmelfarb Health Sciences Library Environmental and Occupational Health Research Guide and the “Grey Matters” tool to identify a comprehensive list of sources for this search. (Levett, 2022; Grey Matters: a practical tool for searching health-related grey literature | CADTH [Internet], 2018) Additionally, the search strategy used, number of results returned for screening, and number of documents included for each source was documented. (Paez, 2017) (Supplement B) Wherever possible and applicable, the grey literature search strategy was consistent across sources.

#### 2.1.2. Study selection

We included primary reports or systematic reviews of frameworks, tools, or templates for making decisions or formulating recommendations, or for priority-setting of interventions or exposures in public health. By employing an inclusive search strategy, we aimed to identify a comprehensive set of frameworks within the subset of environmental and occupational health. We excluded sources that did not have relevance to EOH decision-making, evaluated the effectiveness of specific EOH interventions, or described a framework that is focused exclusively on economic analyses, evidence appraisal, risk assessment, or hazard identification. If multiple documents reported on the same framework, exact duplicates were excluded and duplicated reports of the same framework were only included once, unless they reported on unique criteria or detailed judgements not found in other publications or sources. Two reviewers (EJ, JK, NP, ES) independently screened each title, abstract, and full text in duplicate. At all stages of the review, disagreements were resolved through discussion or by consulting a third reviewer.

#### 2.1.3. Data management and abstraction

Search results were exported to Covidence (Covidence systematic review software, Veritas Health Innovation, Melbourne, Australia. Available at www.covidence.org) to remove duplicates, screen sources, and document reasons for exclusion at the full-text stage (Fig. 1). We used Google Forms to develop and pilot a standardized abstraction tool (Supplement C). If any data items were missing from or ambiguous in the published (or public) framework, we abstracted and analyzed the available information at face value, making as few assumptions as possible about the intent of the framework developer.

Using the piloted tool, we abstracted data from the included sources such as the publication year and venue, study design, geographical location, topic(s), characteristics of the developer(s), intended user(s) and audience, funding sources, development methodology, limitations, decision criteria and signaling questions, use and risk of bias assessment of supporting evidence, and intended application of the recommendations (Supplement C). Abstraction was performed by one researcher and reviewed by a second (EJ, JK, NP, ES). We did not perform a quality appraisal of the frameworks, as this review is concerned with identifying all relevant criteria for EOH decision-making irrespective of the quality of the studies in which they are reported.

#### 2.1.4. Data coding & analysis

**Fig. 2.**
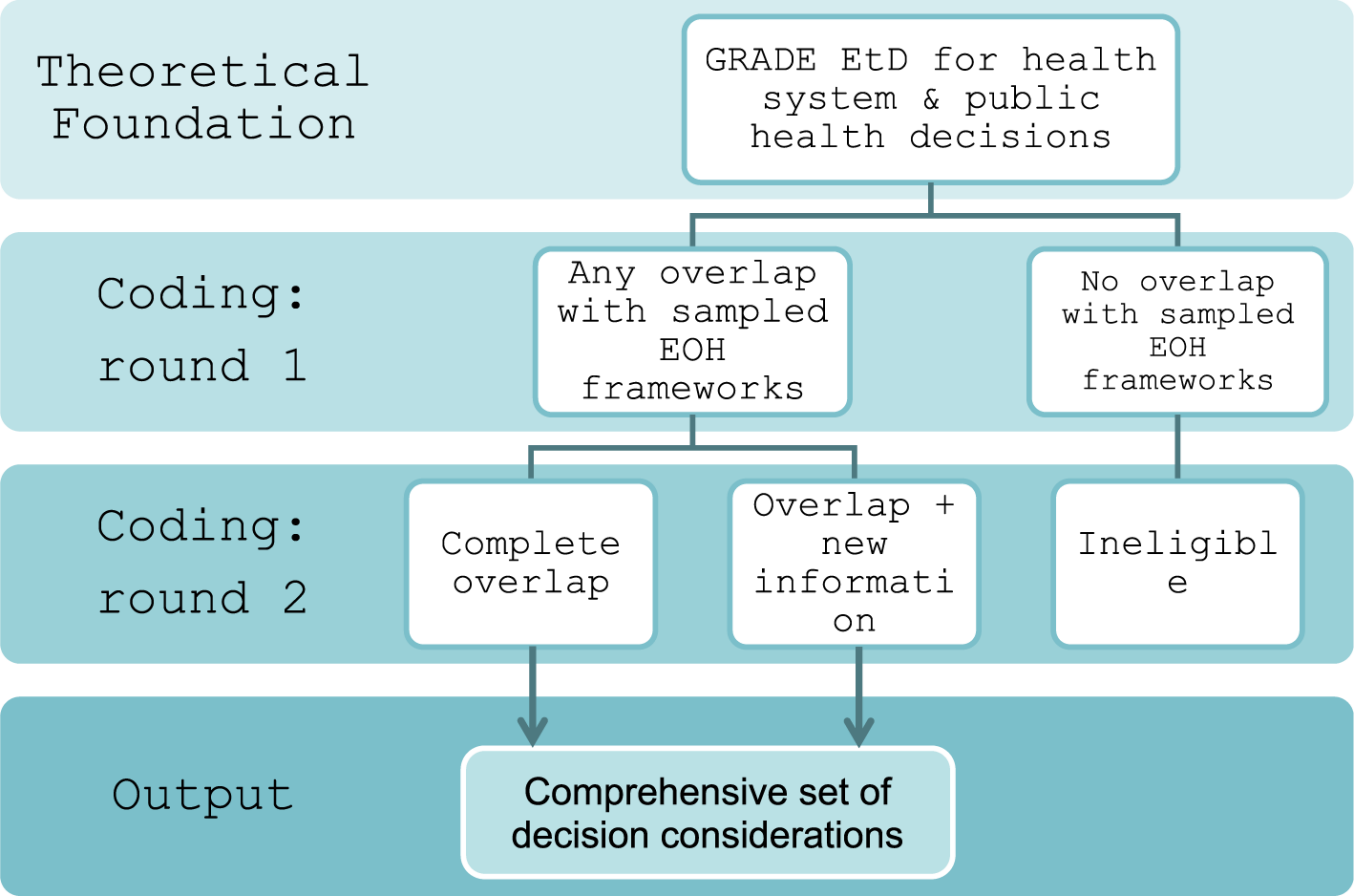
Illustration of deductive coding process for all discovered EOH decision factors.

We conducted a narrative analysis using textual descriptions and tabulation to systematically describe the characteristics of the included frameworks. For the individual decision considerations abstracted from the included frameworks, we designed a coding strategy to map abstracted considerations onto the decision criteria presented in the *GRADE EtD for health system and public health decisions*. With this approach, we aimed to identify (a) the potential existence of an EOH decision framework that would make tailoring of the GRADE EtD framework redundant, (b) whether any EOH frameworks introduce a theme that is not part of the GRADE EtD, thereby necessitating its extension, and/or (c) whether components within the EOH frameworks could inform tailoring of the GRADE EtD to the specifics of EOH decision-making contexts (Fig. 2).

Each of the GRADE EtD framework assessment criteria (Problem, Desirable effects, Undesirable effects, Certainty of evidence, Values, Balance of effects, Resources required, Cost effectiveness, Equity, Acceptability, Feasibility) served as a code that could be applied to the abstracted decision considerations (Gray and Cohen, 2012) (Supplement E). Examples of this process are presented in Table 1.

During the initial phase of coding, two members of the research team (PW, ES) independently applied codes to a pilot set of 100 abstracted decision considerations. Any discrepancy in application of the codes was counted as a disagreement and discussed to facilitate increased consistency in coding decisions between the researchers. After the initial calibration exercise, remaining considerations were coded by one researcher and reviewed by a second (PW, ES). In cases of disagreement, the reviewing researcher could apply additional codes to a consideration, but existing codes were not removed. We selected this inclusive approach to coding to account for multiple plausible interpretations of the same information.

We applied as many of the codes as could reasonably be related to each decision consideration, hereafter referred to as “items.” We prospectively defined an “item” as the smallest unit of each framework, meaning the most detailed or granular description of a decision criterion (Table 2). Some items encompassed multiple EtD criteria and vice versa; rather than selecting a single, best fitting code for each item, we assigned as many codes as needed. Items were also labeled as “guidance” if they were normative statements or statements about the process for making decisions (e.g., “minimize harm to the general population,” “protect human rights and individual autonomy”); items were coded as “no code” if they had no plausible correlate within the assessment criteria or detailed judgements of the GRADE EtD framework.

During the second phase of the coding process, three members of the research team (PW, RM, ES) reviewed and discussed all items that were coded to each of the GRADE EtD assessment criteria, including items for which a code was not identified. Additional codes were applied to items that may inform guidance about implementing or operationalizing the GRADE EtD in EOH contexts, inform tailoring of the GRADE EtD scope and context criteria, or comprise new judgements for a GRADE EtD assessment criterion to which they were coded in the first round. These items, collectively coded as “unique,” were de-duplicated and organized into a consolidated set of items for further refinement through a Delphi process.

We summarized the coding applied to the identified detailed judgments with descriptive statistics (e.g., frequencies, percentages) using R Statistical Software (v4.1.2; R Core Team 2021). (Popay et al., 2006; Campbell et al., 2020; McHugh, 2013) The data set and code we used for this analysis are available at: https://github.com/esenerth/GRADE-EOH-EtD.

### 2.2. Delphi study

During the second phase of the project, we selected an electronic Delphi process to elicit additional information from subject matter experts because it prevented more outspoken or apparently senior participants from dominating a group discussion and therefore influencing others, permitted broader geographic representation than an in-person format, accommodated inclusion of diverse stakeholder perspectives, and provided an established method for reaching consensus.

#### 2.2.1. Study design

Results of the systematic review and narrative synthesis underwent further analysis through structured stakeholder input to identify any EOH decision considerations that are not evident in the published or gray literature. Additionally, panelists were instructed to recommend reorganization, consolidation, and/or rewording of the decision criteria and detailed judgments as the basis for development of an integrated decision framework that will be applicable to EOH. This prospective Delphi study has been conducted and reported according to published guidance. (Hasson et al., 2000; Grime and Wright, 2016; Jüngeretal., 2017; Linstone and Turoff, 1975).

#### 2.2.2. Stakeholder panel

Stakeholders were recruited by the research team based on a matrix describing characteristics of anticipated users of an EOH decision framework and/or consumers of EOH recommendations. (Hasson et al., 2000) Prospective panelists received an invitation via email detailing the objectives, anticipated process, and timeline of the study. The panel was appointed after ensuring consent to participate and balanced representation across gender, geographical settings, institutional contexts, and topical expertise. Prior to convening the panel, we collected information on intellectual and financial relationships from participants and did not identify any conflicts that would necessitate recusal for any portions of the process.

#### 2.2.3. Study procedures

Panelists received instructions and survey materials via email; the survey was developed and fielded in Excel (Version 16.67; Microsoft, 2022). (Supplement F) Panelists rated criteria and detailed judgements abstracted and consolidated from the systematic review and narrative synthesis, along with criteria and detailed judgements from the GRADE EtD framework as presented in GRADEPro (gdt.gradepro.org), on a 7-point Likert scale in the following domains: agreement with inclusion of the consideration (1 = strong disagreement; 7 = strong agreement), agreement with the wording of the consideration (1 = strong disagreement; 7 = strong agreement). The questionnaire also included multiple opportunities for free text comments: suggestions for revision of the wording, suggestions for additional guidance, other comments on the criteria or detailed judgements, and addition of any new criteria or detailed judgements. Questionnaires were completed independently, and panelists were not provided with any information about how others had voted in the prior round. (Supplement G).

Panelists were recruited based on a purposive sample developed by the research team and through “snowball sampling” of invitees. We aimed to recruit between 15 and 25 total panelists comprising target users of the GRADE EtD for EOH, participants with expertise in GRADE methods, and participants with expertise in several subspecialties of environmental and occupational health. Delphi literature recommends engaging between 10 and 18 participants per panel for optimal group dynamics and achieving consensus within a feasible number of rounds. (Veugelers et al., 2020) The first round of the Delphi exercise began in March 2022 and lasted 14 days. Participants were sent up to three reminders to complete the questionnaire. The second round began in June 2022 and used a modified questionnaire based on iterative feedback and consensus during Round 1. This round also lasted 14 days, with up to three reminders.

#### 2.2.4. Analysis plan

Survey responses were anonymized by one member of the research team (ES) and processed by three members of the research team (ES, PW, RLM) during a series of virtual meetings. The primary outcome was attainment of consensus on the inclusion and wording of each criterion and detailed judgment. Responses, including panel demographic characteristics, were analyzed using descriptive statistics: counts and percentages, mean (standard deviation), median (interquartile range) and range. (Holey et al., 2007) Items that received a median rating of > 6.9 automatically advanced to the next round; items that received a median rating of < 4.9 were removed. Items with median scores in between these values and/or items demonstrating large variability in rating (i.e., IQR > 2) were discussed during the consensus meetings, including qualitative analysis of free text comments. These were coded into four categories: “scope (item should be more specific to environmental health, or subtypes of interventions or other policies), redundancy (addressed within or duplicated by another criterion or item), new (new decision consideration proposed by the respondent), and clarity (the content of the item is unclear to the respondent).

#### 2.2.5. Ethics

This project did not involve any data collection from human subjects or biological specimens. We collected and analyzed existing data from published or public sources. According to the Hamilton Integrated Research Ethics Board (HiREB), this project does not require ethics review and is granted a waiver from the TCPS2 (2018) Article 2.5 as of January 24, 2022. Findings may be published and/or presented as quality improvement information.

## 3. Results

### 3.1. Systematic review

#### 3.1.1. Search results

The search of published literature yielded 5,420 records for consideration. After removing duplicates (n = 224), we reviewed the titles and abstracts of 5,196 records and excluded 4,918 that did not meet the criteria for inclusion. We reviewed the remaining 278 full texts and excluded another 255. The most common reason for exclusion was that the study did not present a decision-making framework (n = 217); other excluded records presented hazard identification frameworks (n = 29) or evidence appraisal frameworks (n = 6). (Supplement D) The search of gray literature yielded 835 documents from 31 different organizations. After assessment against the inclusion criteria, 22 published reports of frameworks and 16 frameworks from the gray literature were advanced to data abstraction (Fig. 1).

#### 3.1.2. Framework characteristics

Of the 38 included frameworks, 18 (47.4 %) were developed by or for government agencies, 14 (36.8 %) were developed in academic settings, 5 (13.2 %) were developed by non-governmental organizations (NGOs), and 1 (3 %) was developed by industry. The most common topic addressed by the frameworks was public health (n = 11). (Rehfuess et al., 2019; Health Quality Ontario. Health Technology Assessment (HTA) Methods and Process Guide, v2.0 [Internet], 2018; SIGN Executive, 2019; CADTH, 2020; WHO, 2020; CDC, 2022; Carande-Kulis, 2012; González-Lorenzo et al., 2015; Krahn et al., 2018; Sampietro-Colom, 2015; Siu, 2021) Other topics included water management (n = 7), (Bernstein, 2015; Garfi and Ferrer-Marti, 2011; Marazzi et al., 2020; Naman and Gibson, 2015; Pang et al., 2017; Song and Kim, 2021; Williams and US EPA, 2021) chemical alternatives assessment (n = 5), (Dorman et al., 2022; Malloy et al., 2013; Mitchell et al., 2013; Moermond et al., 2012; Perez et al., 2017) waste management or sanitation (n = 4), (Garfi and Ferrer-Marti, 2011; Naman and Gibson, 2015; Adar et al., 2020; Malekpour et al., 2013) workplace exposures (n = 4), (Deveau et al., 2017; Felknor et al., 2019; Morley et al., 2017; Ramos, 2018) site remediation (n = 2), (Burger and Gochfeld, 2020; Cappuyns, 2016) disaster or emergency management (n = 2), (Kayman and Logar, 2016; Mullen et al., 2020) and emissions (n = 1). (He and Hung, 2012) Four reports of frameworks did not specify a topic (US EPA, 2013; Persson, 2016; Resnik et al., 2018; Woods et al., 2016) and two addressed both water management and sanitation (Table 3). (Garfi and Ferrer-Marti, 2011; Naman and Gibson, 2015) The frameworks that contributed the largest number of items to this analysis originated from government (n = 275) and academic (n = 241) settings, and addressed water management (n = 133) and chemical alternatives assessment (n = 113) (Table 4). Although public health was the most common framework topic, addressed by 29 % of frameworks, public health frameworks contributed a minority of items to the analysis.

#### 3.1.3. Coding results

**Fig. 3.**
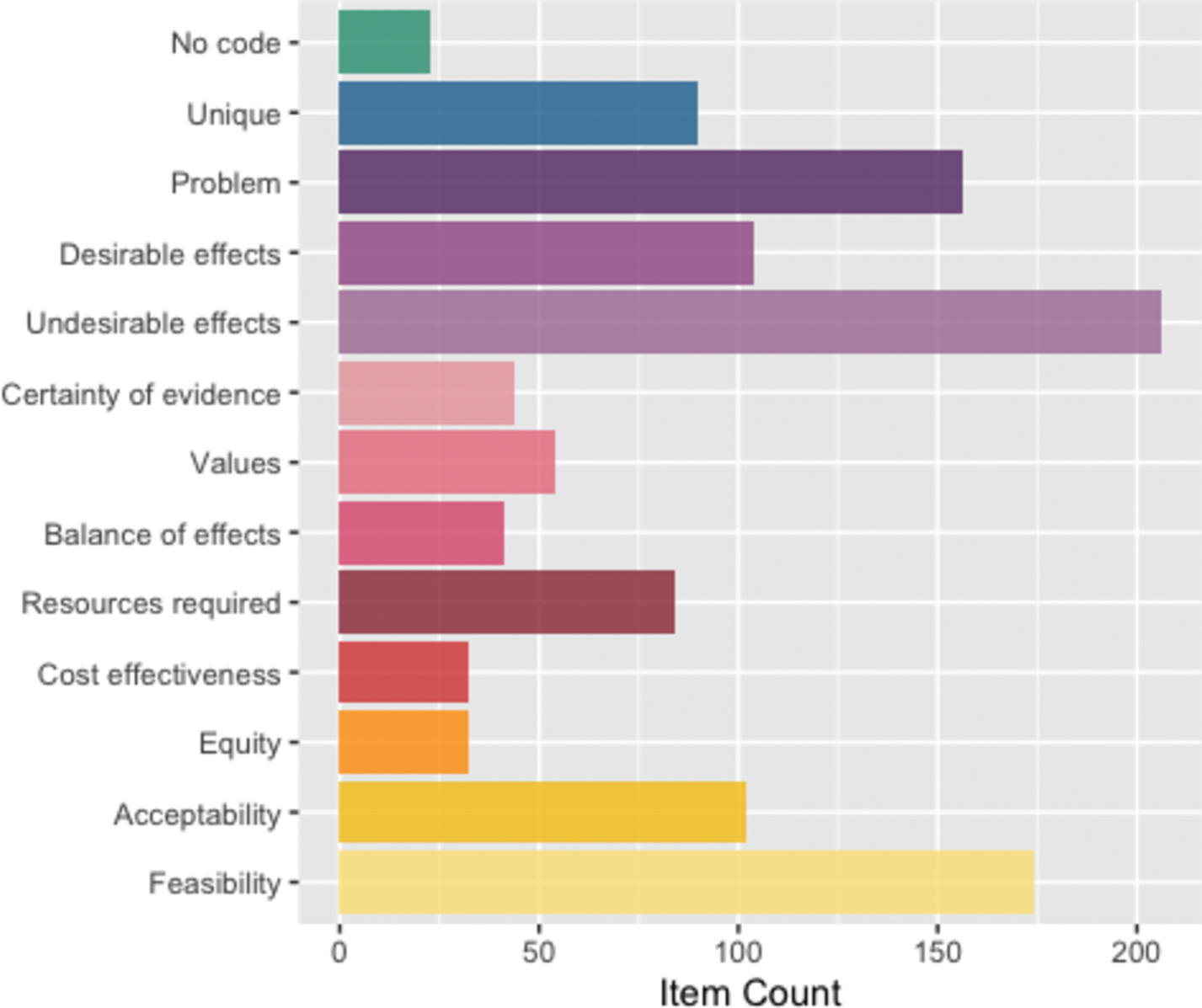
Summary of coding decisions for all discovered EOH decision factors.

**Fig. 4.**
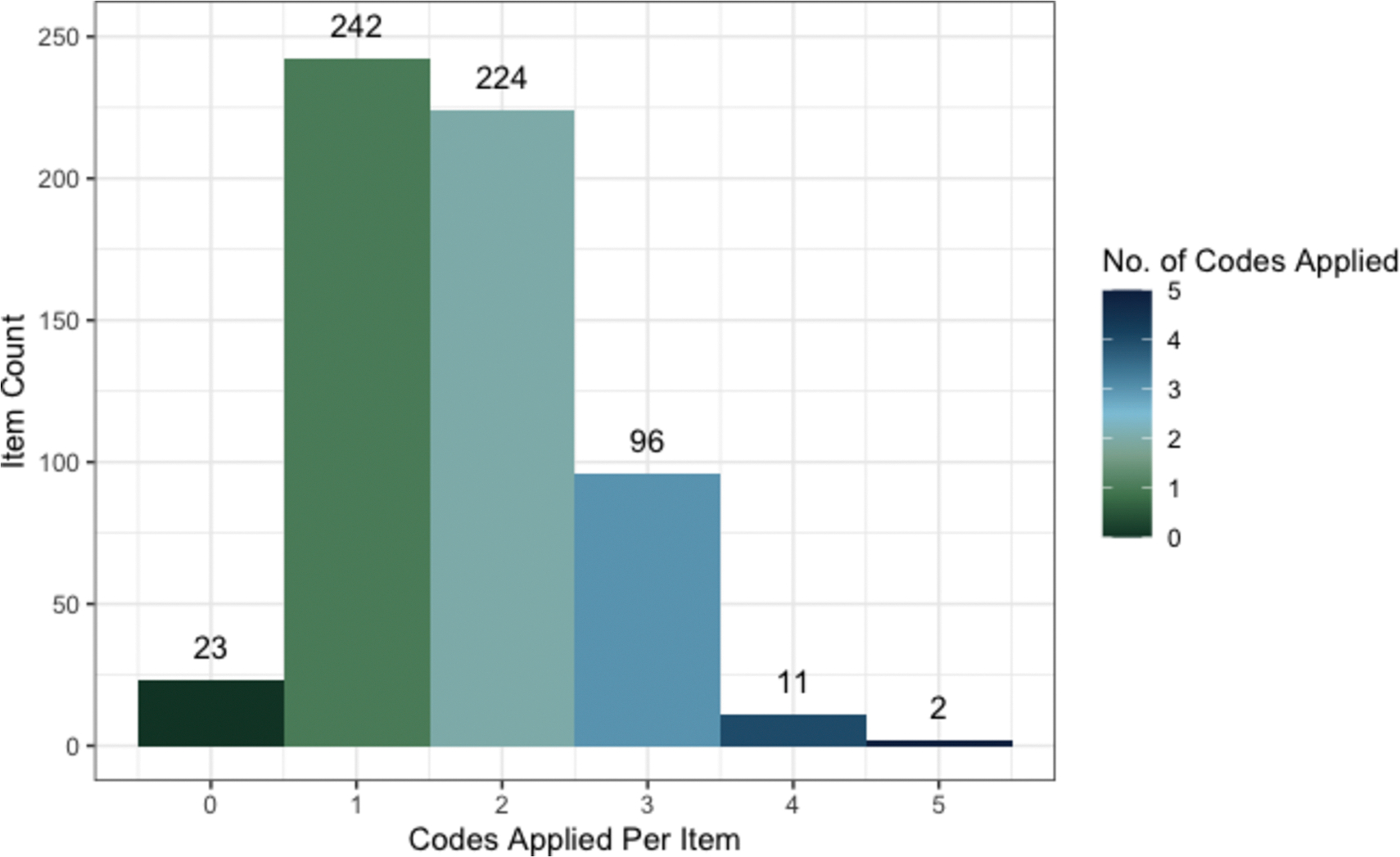
Count of codes applied to each discovered EOH decision factor.

Researchers applied identical codes to 90 percent of the items during piloting. Subsequently, 89.3 percent of the items were coded identically. In the first round of coding, out of the 560 identified items abstracted from the included frameworks, 206 (36.8 %) were coded to “Undesirable effects”, 174 (31.1 %) were coded to “Feasibility”, 156 (27.9 %) were coded to “Problem”, 104 (18.6 %) were coded to “Desirable effects”, 102 (18.2 %) were coded to “Acceptability”, 84 (15.0 %) were coded to “Resources required”, 54 (9.6 %) were coded to “Values”, 44 (7.9 %) were coded to “Certainty of evidence”, 41 (7.3 %) were coded to “Balance of effects”, 32 (5.7 %) were coded to “Equity”, and 32 (5.7 %) were coded to “Cost effectiveness” (Fig. 3, Table 4). Most of the items had two or more codes applied. (Fig. 4).

Twenty-three identified items were designated as “no code,” meaning they had no plausible correlation with any of the criteria nor of the detailed judgments of the GRADE EtD framework for health system and public health decisions (Fig. 3). One framework was excluded from further analysis because it provided criteria for assessing access to health services rather than criteria for health decision-making; this framework contributed 19 items to the “no code” category. (Song and Kim, 2021) This framework could also reasonably have been excluded during the screening phase. The other 4 items coded as “no code” were identified in two frameworks and were excluded from further analysis because they described criteria for assessing the decision-making process rather than criteria for assessing alternatives. (Dorman et al., 2022; Malekpour et al., 2013) Ultimately all the “no code” items were excluded from further analysis for the reasons described above. Our sample did not include any items that were completely distinct from the GRADE EtD framework criteria for health system and public health decisions.

**Fig. 5.**
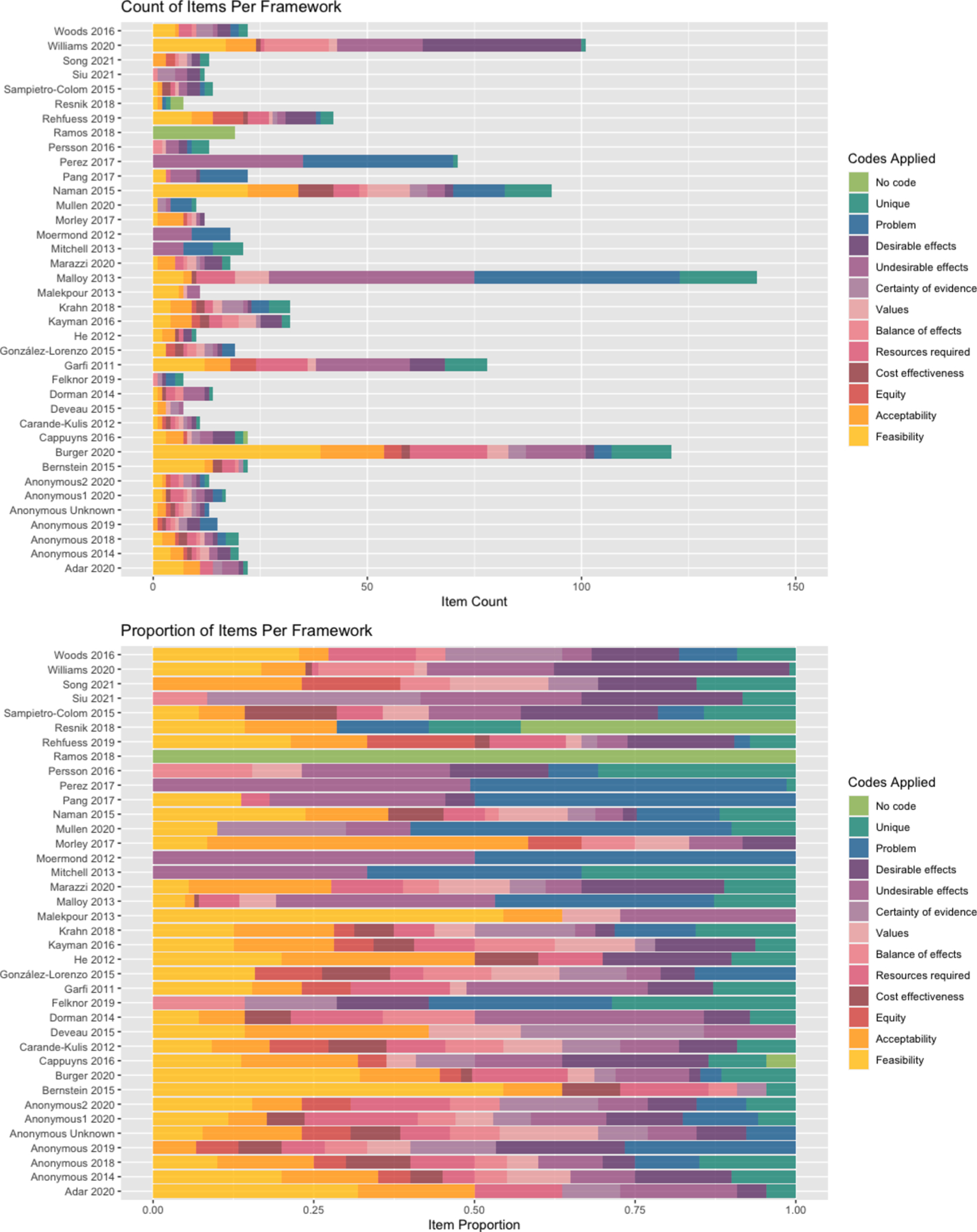
Count and proportion of codes applied to discovered EOH decision factors per framework.

During the second phase of coding, we applied additional codes to denote items that are aligned with one or more of the GRADE EtD criteria, but also contribute additional breadth or detail to the guidance (n = 2), scope and context (n = 40), or detailed judgements (n = 62) of the GRADE EtD criteria to which they were coded in the first round (Table 5). In aggregate, these items are coded as “Unique” (Fig. 2). We observed the greatest number of unique items coded to “Problem” (n = 31) and “Resources required” (n = 25) (Table 5). Of 38 included frameworks, 28 of these contributed items that were subsequently coded as unique (Fig. 5).

We observed variability in the total number of items abstracted from each of the frameworks (Fig. 5). The frameworks with the greatest contribution of items were developed to address water management, chemical alternatives assessment, sanitation, and site remediation. (Garfi and Ferrer-Marti, 2011; Naman and Gibson, 2015; Williams and US EPA, 2021; Malloy et al., 2013; Perez et al., 2017; Burger and Gochfeld, 2020) These frameworks described decision considerations that are aligned with several of the GRADE EtD criteria (feasibility, desirable and undesirable effects, priority of the problem) but expressed them in a more granular form (e.g., carcinogenicity, genotoxicity, developmental toxicity, reproductive toxicity, and endocrine disruption disaggregated into separate considerations rather than a single judgment about “toxicity”).

**Fig. 6.**
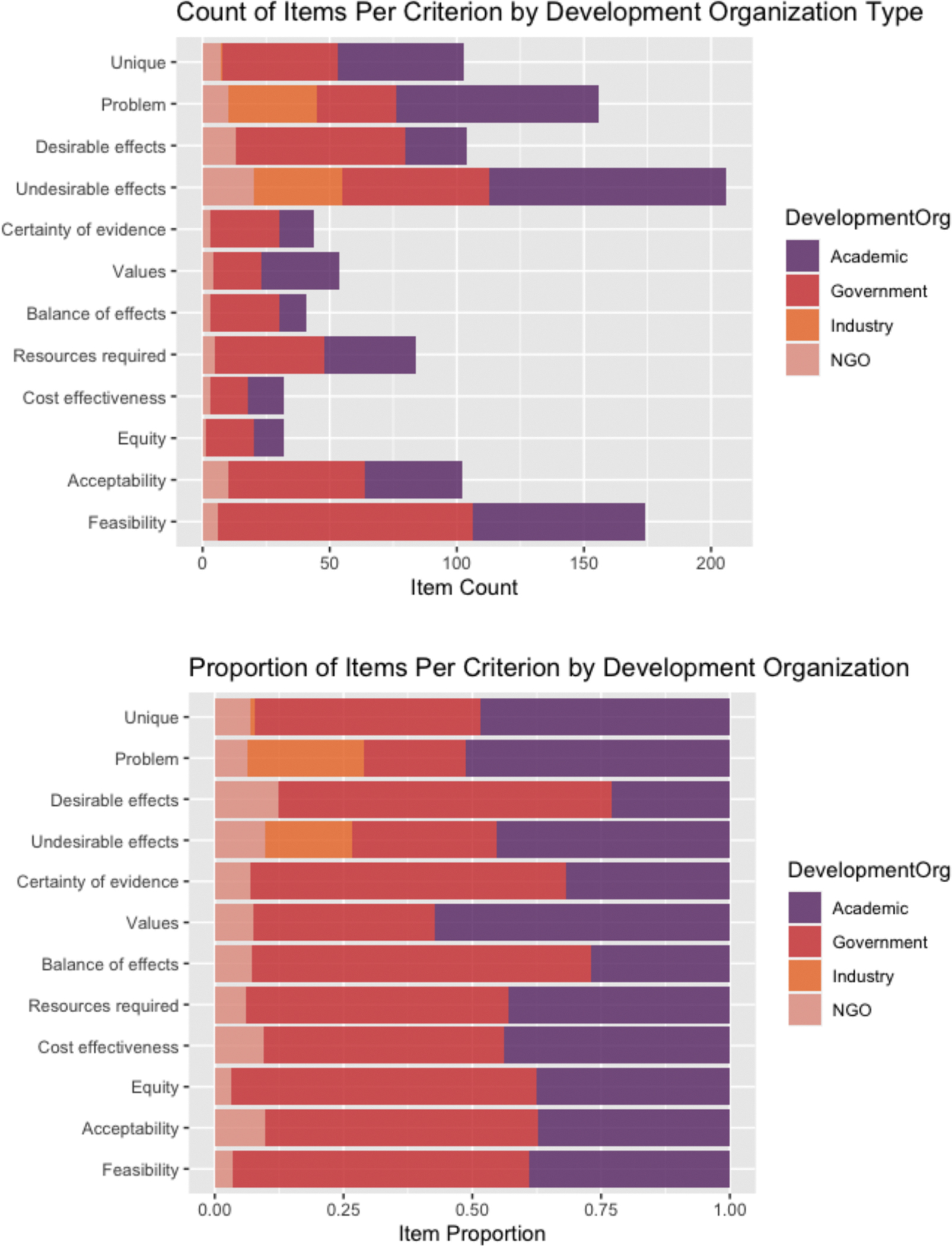
Count and proportion of codes applied to discovered EOH decision factors by type of organization that developed the framework.

**Fig. 7.**
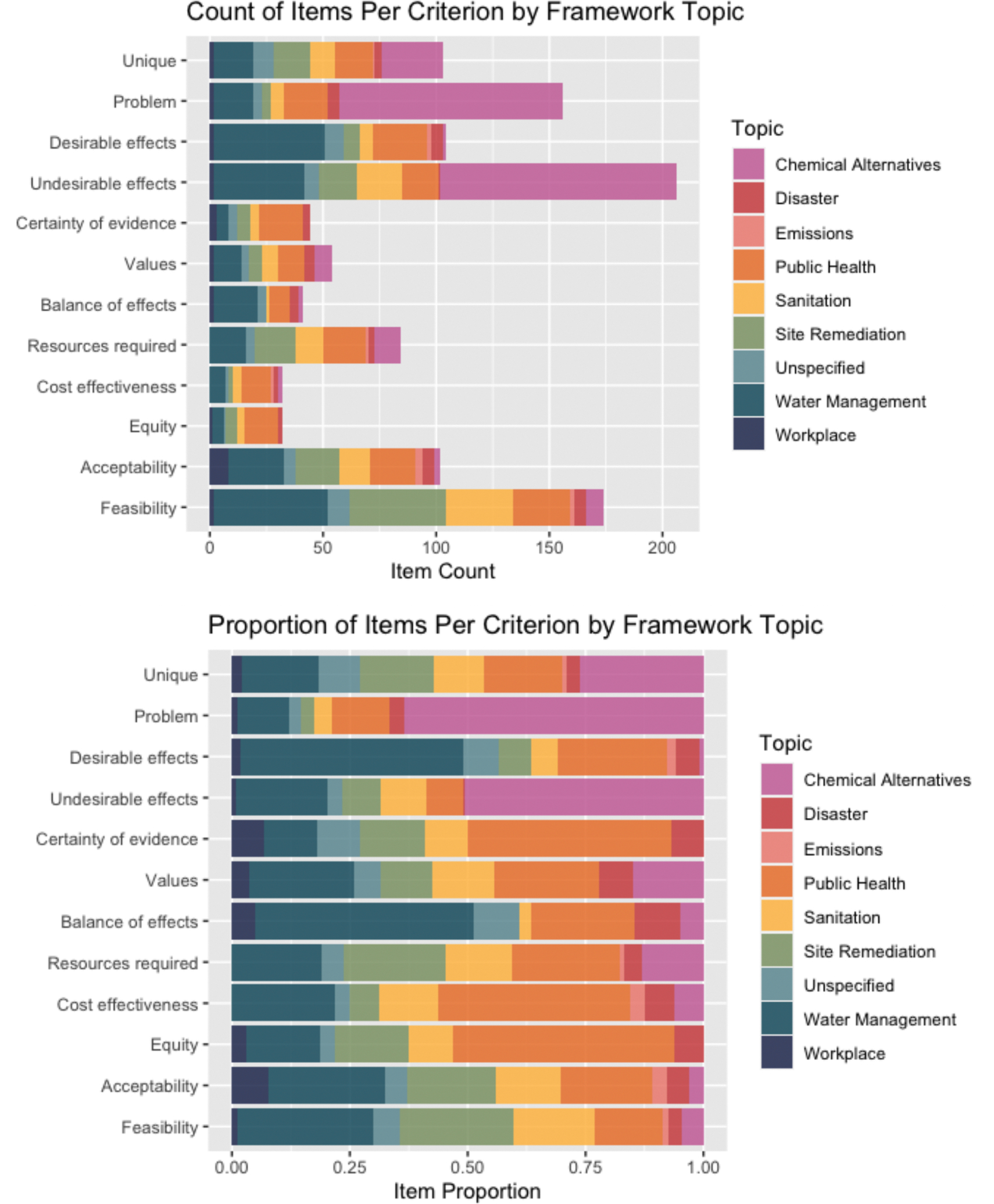
Count and proportion of codes applied to discovered EOH decision factors by framework topic.

Analysis of the distribution of items stratified by framework characteristics shows that the proportion of identified framework items coded to each GRADE EtD criterion varies by both the development organization and topic of the framework (Table 3). The framework developed by industry primarily concentrated on the “Problem” and “Undesirable effects” criteria. Frameworks developed by or for government agencies had the greatest density of items coded to “Feasibility.” Finally, frameworks developed in academic and government settings collectively contributed most of the unique items (Fig. 6). By topic, chemical alternatives assessment frameworks had the greatest relative density of items coded as “Problem” and “Undesirable effects” (Fig. 7). The narrow focus of both industry and chemical alternative frameworks is likely driven by Perez 2017, which contributed items to both categories, and Malloy 2013, which addressed chemical alternatives assessment from an academic perspective. (Malloy et al., 2013; Perez et al., 2017) Public health frameworks had the most uniform distribution of items across the EtD criteria.

Based on the criteria that comprise the GRADE EtD, the frameworks identified by our review focused on assessing the undesirable effects of exposures, feasibility of alternatives, and magnitude or priority of the problem under consideration. The frameworks gave comparatively less attention to assessing the impact on equity and cost effectiveness of alternatives. We observed variability in the distribution of items related to each EtD criterion both in aggregate and when we stratified the data based on framework characteristics.

### 3.2. Delphi study

**Fig. 8.**
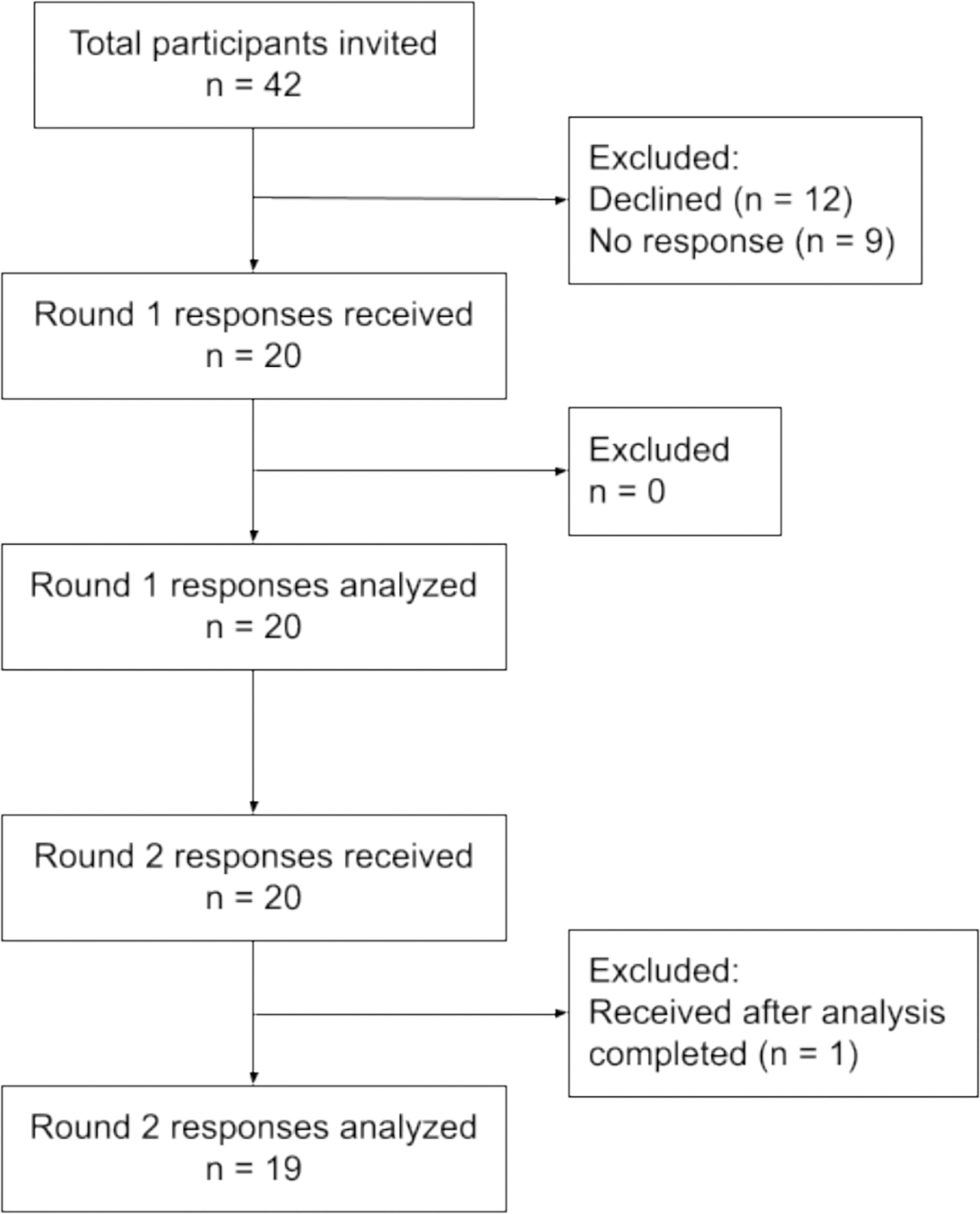
Participation in the Delphi process.

Out of a total of 42 invitations, 21 participants (50 %) accepted the invitation and provided consent. One participant withdrew before completing the first round of rating. We received 20 complete responses to the round one survey and 20 complete responses in round 2. However, one round 2 response was received after feedback was compiled and processed, so was not analyzed (Fig. 8). Demographic characteristics of the panel surveyed in each round are presented in Table 6. The demographic composition was similar in rounds one and two, with roughly equal distribution of male and female participants and roughly equal representation of academic or research and government or regulatory settings. Most participants in both rounds are from North America (mean 46 %); Europe, Asia, Australia, Africa, and South America were also represented on the panel.

**Fig. 9.**
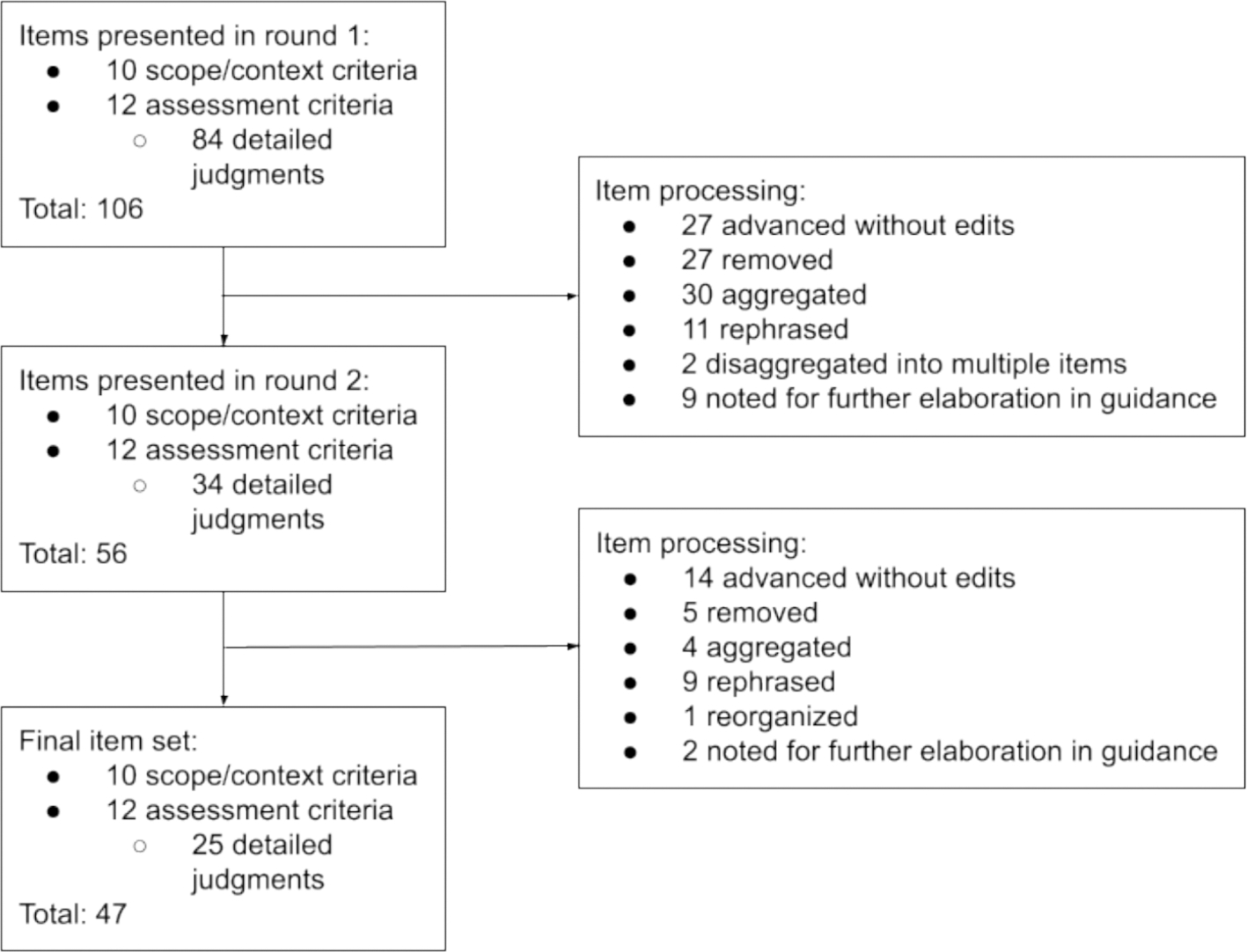
Disposition of items presented for rating.

We presented a total of 106 items for rating in round one: 10 scoping criteria, 12 assessment criteria and 84 detailed judgments that may inform decisions about the main criteria. Based on panel free-text feedback and pre-specified consensus thresholds, 27 items were advanced from round one without any edits, including all the assessment criteria, and 27 items were dropped from the set. Other items were aggregated together (n = 30), rephrased (n = 11), or disaggregated into multiple items (n = 2) for re-rating in round two. Finally, we noted nine items that require further elaboration in the guidance for implementation of the EOH GRADE Evidence-to-Decision framework. Respondents did not propose any new decision considerations in round one. (Table 7, Fig. 9).

In round two, we again presented 10 scoping criteria and 12 assessment criteria for re-rating by the panel. The detailed judgments disaggregated from the assessment criteria were revised, reduced, and consolidated into 34 items for re-rating. The round two questionnaire contained 56 total items for re-rating. Based on panel free-text feedback and pre-specified consensus thresholds, 14 items were advanced from round two without any edits, and 5 items were dropped from the set. Other items were aggregated together (n = 4), rephrased (n = 9), or reorganized into a different grouping of decision considerations (n = 1). Finally, we noted two additional items that require further elaboration. Respondents did not propose any new decision considerations in round two. (Table 7, Fig. 9).

### 3.3. Themes

Through this iterative process of collecting, de-duplicating, reorganizing, clarifying, and consolidating EOH decision considerations for further integration into a tailored EtD framework for EOH decision-making, we observed several themes as follows:

#### 3.3.1. Accounting for broad, undefined constituencies in decision-making

Several frameworks in our sample explicitly referenced consideration of local community views and norms, interaction with various levels of government, broadly shared values in society, and values that tend to be minimized through methods used in traditionally hierarchical decision-making processes. (Rehfuess et al., 2019; CADTH, 2020; Sampietro-Colom, 2015; Garfi and Ferrer-Marti, 2011; Naman and Gibson, 2015; Williams and US EPA, 2021; Morley et al., 2017; Cappuyns, 2016; Kayman and Logar, 2016; Mullen et al., 2020; He and Hung, 2012; US EPA, 2013; Persson, 2016) EOH decisions frequently affect large populations and diverse stakeholders, which requires decision-makers to define the constituency for a recommendation and make determinations about which stakeholder values are considered and weighted. For example, prioritizing one problem over another may involve an implicit judgment about the values of the population.

Delphi panelists emphasized the importance of specifying the stakeholders who are the subject of consideration (e.g., when making a judgment about whether an intervention is acceptable to key stakeholders, “stakeholders at different levels may have various concerns on the intervention impacts, options, consequences, etc., try to specify the support from stakeholders with more details such as types.”). They also questioned whether the values, engagement, or awareness of a population should factor into decision-making about exposures or interventions to mitigate exposures with established harmful effects (e.g., “Thinking about hazardous exposures, I’m not sure this should be factored in.”).

#### 3.3.2. Consent

In scenarios where broad awareness of an EOH problem and decision alternatives cannot be assumed, consent may be a relevant consideration across several criteria. For example, when assessing whether a problem is a priority, frameworks in our sample described community involvement and advocacy as an indicator of urgency that could inform prioritization of certain questions or problems over others. When assessing acceptability, the frameworks described compulsion, coercion, and individual autonomy as considerations. When assessing feasibility, frameworks were concerned with whether or not the legal and regulatory context could provide an enforcement mechanism for a recommendation or threshold.

#### 3.3.3. Timing

Timing is considered when assessing priority of the problem (e.g., bioaccumulation potential or persistence in the environment of an exposure), the resources required (e.g., age of equipment, institutional knowledge of workers), and the desirable and undesirable effects of different options (e.g., time for a potentially beneficial intervention to reach full effectiveness compared to an alternative). Specifically, EOH frameworks often balance short-term undesirable effects (e.g., increased stress) and long-term desirable effects (e.g., reduction in community incidence of emphysema). Conclusions may differ depending on which time point is considered the most important.

#### 3.3.4. The precautionary principle

Several frameworks in our sample were concerned with operationalizing the precautionary principle in EOH decision-making. For example, the likelihood of false negatives versus false positives as a consideration as part of the desirable and undesirable effects of alternatives (e.g., “it is more important to avoid false negatives than false positives,” “timing is at least as important as being right”). The precautionary principle is also present in detailed judgments about acceptability and values, which facilitate assessment of the risk tolerance of various stakeholder groups through the consideration of relevant outcomes (i.e., risk acceptance by placing low value on possible undesirable health outcomes, or risk aversion by placing high value on possible undesirable health outcomes).

#### 3.3.5. Social and environmental justice

Delphi panelists suggested broadening the equity criterion to include consideration of the geographical dimensions of an intervention or exposure and issues of social justice/injustice (e.g., “There are environmental exposures that impact communities where the community has no say, e.g. wind turbine facilities. This can be viewed as social injustice. Furthermore, in this scenario, a landowner may directly benefit because the turbine is on their property, a neighbor who lives closer to the turbine may get nothing.”) Social and environmental justice considerations were also apparent in our sample of EOH decision frameworks (e.g., “Social justice and equality: How is social justice and/or equality addressed? What is the duration of remedial works and are there issues of intergenerational equity? Are the impacts/benefits of works unreasonably disproportionate to particular groups?”).

#### 3.3.6. Barriers to operationalizing the framework

The EOH decision frameworks in our sample tended to forefront granular and context-specific decision considerations, often in the form of highly specific lists. For example, several frameworks provided extensive lists of stakeholder perspectives to consider when assessing acceptability, resources to consider when assessing the required resources, and toxicities to consider when assessing the undesirable effects of an exposure and priority of the problem. This specificity may be intended to minimize variation in operationalization of the frameworks or provide sufficient guidance to support implementation in a particular decision context, but also may limit their generalizability. The result is a patchwork of EOH decision frameworks that are each applicable to specific contexts, topics, or scenarios, but no prevailing framework of broad utility. (Table 3).

Feedback from Delphi panelists was also focused on barriers to consistent operationalization of the framework. For example, panelists requested static definitions or quantitative thresholds for terms such as “extraordinary” and “important” when these were used to qualify decision considerations (e.g., “Does the problem constitute an extraordinary event?”). These instances were noted for future development of guidance. Panelists also suggested re-wording or reorganization to improve generalizability of considerations that had been abstracted from frameworks developed with a specific context in mind (e.g., “Extent to which funding for intervention is a city or county priority compared with other rivaling priorities” should be revised to remove terms with limited applicability such as “county” and replaced with general terms such as “local.”) Finally, panelists highlighted scenarios where specific detailed judgments would not apply and noted that they were uncertain about how to respond if a judgment was not relevant or applicable to a given scenario.

## 4. Discussion

### 4.1. Statement of the principal findings

Through a systematic review and Delphi process, we have observed that concepts put forward in EOH decision frameworks and by subject matter experts can plausibly be mapped onto the criteria of the GRADE EtD framework. These results suggest that the GRADE EtD framework is applicable for use in EOH decision-making (e.g., determination of an allowable level or threshold of an exposure, alternatives assessment, or recommendation of an intervention to prevent or mitigate an exposure). However, as mentioned in the development of the original GRADE EtD frameworks, some criteria may be more or less relevant depending on the specific decision context.

One key modification in our proposed framework is broadening the scope of the GRADE EtD health equity criterion to include considerations beyond health equity. Unequal distribution of environmental burdens and benefits is a feature of social, political, and economic systems. The natural and built environment, including workplaces, are key sites whereby “resources like knowledge, money, power, prestige, and social connections are transformed into the health-related resources that generate patterns of morbidity and mortality.” (Link and Phelan, 1995) EOH decision-makers may often need to account for both immediate (proximal) and indirect (distal) factors associated with health outcomes. Deliberate consideration of the socio-political and economic context is important for recognizing when an intervention or option may perpetuate or increase inequity, as these conditions are instrumental in shaping patterns of exposure. Failure to consider context may result in “interventions [with targets] that are resistant to change for unrecognized reasons.” (Link and Phelan, 1995) Social context is partially accommodated in other EtD criteria, such as values, acceptability, feasibility, but also should be accommodated in equity to account for disparities that are immediately connected to health outcomes, as well as further upstream from these outcomes.

Other assessment criteria underwent minor modification through changes to their composite detailed judgments. These changes incorporate concepts that were surfaced from the EOH literature and confirmed by the Delphi process. Examples are consideration of irreversibility when judging the priority of a problem and consideration of time span for sustainability when assessing feasibility. Finally, detailed judgments informing the “Resources” and “Cost-Effectiveness” criteria were simplified and consolidated based on feedback from the panel that more granular or specific prompts were difficult to interpret and had limited applicability. The GRADE EtD framework for EOH and accompanying guidance are presented in the companion publication to this manuscript (**CROSS REF – TK**).

### 4.2. Strengths and weaknesses of the study

This work is intended to support an exhaustive and transparent process for development of an EtD framework that is suitable for EOH decision-making. We used a rigorous methodology to systematically identify and screen decision-making frameworks, adopting an inclusive approach to capture all decision factors that could be relevant to EOH. Our analysis is grounded in an established framework, the *GRADE EtD for health system and public health decisions*. Through narrative synthesis, we were able to summarize and analyze factors and criteria that are not readily explored using other techniques. For example, we used thematic analysis to identify relationships between the sampled frameworks and the GRADE EtD for health system and public health decisions.

This systematic review was limited to frameworks that exist in the published or public domain and does not include proprietary, confidential, or undocumented frameworks that may be used for EOH decision-making. This potentially reduced the size of our sample, and the perspectives represented therein. Additionally, restricting our sample to frameworks that are available in the English language may have excluded otherwise eligible documents. Judgments about inclusion or modification of EOH decision criteria made by this research team are inherently subjective and may not be replicable. Further, our interpretations of ambiguous EOH decision criteria are informed by associated written material made available by framework developers, but also involve subjective judgment. Statistical analysis of the association between framework characteristics and decision considerations is also limited by our sample size. These results are intended to be hypothesis-generating rather than conclusive. Further development and validation of a proposed EtD framework for EOH is outside the scope of this study.

Further, we have minimal certainty in the replicability of the Delphi study results, meaning that another panel may receive the same questionnaires and provide different responses. Additionally, our panel is not completely representative of intended users; some geographical regions, agencies, and perspectives are not represented because of logistical constraints. Although an important feature of the Delphi process, attainment of consensus does not necessarily mean that a single correct opinion or judgment has been discovered. Instead, we have identified concepts that one group considers to be important for EOH decision-making. Finally, decisions made by the research team about how to implement vague or contradictory feedback from the panel are inherently subjective.

### 4.3. Relation to other studies

This work extends ongoing efforts to develop a systematic approach to collect, synthesize, and evaluate scientific evidence linking environmental exposures to health outcomes. (Morgan et al., 2018; Morgan et al., 2016; Woodruff and Sutton, 2014; Norris et al., 2021; Morgan et al., 2019) We have aimed to address the juncture where scientific evidence is used to inform policy, regulation, and other, similar decisions in EOH.

Additionally, our findings contribute to a growing body of literature on usage of the GRADE EtD frameworks.(Norris et al., 2021; Dewidar et al., 2023; Meneses-Echavez et al., 2023; Stadelmaier et al., 2022; Morgano et al., 2022; Friesen et al., 2022; Piggott et al., 2022; Barnabe et al., 2021; Piggott et al., 2021) We found that most of the EOH decision considerations in our sample were related to more than one of the GRADE EtD criteria. This finding reflects three forms of ambiguity: multiple plausible interpretations of the EOH framework terminology, lumping of multiple concepts within one EOH decision consideration, and the inter-relatedness of the GRADE EtD criteria themselves. Context-specific tailoring of the GRADE EtD detailed judgements and guidance has the potential to provide additional clarity and structure to support operationalization of the framework.

### 4.4. Meaning of the study: Possible explanations and implications for stakeholders

Abstracted contextual information may explain some of the observed similarities and differences between EOH decision frameworks and the GRADE EtD. Many frameworks in our sample were developed to address specific topics and decision types (e.g., platform decommissioning), and are thus inherently specific to the perspective taken by their developers. Examining the distribution of detailed judgements across types of developers and topics provides insight into which criteria are most frequently considered or consistently deemed relevant by EOH stakeholders from a variety of perspectives. Differences we observed in the detailed judgments across perspectives could also be explained by varying degrees of familiarity with the GRADE EtD framework among EOH framework developers. When stratified by framework characteristics, our data describe trends in alignment with the GRADE EtD and in turn, identify which types of decision-makers may be more or less amenable to adopting an EOH EtD framework.

During both phases of the project, we also aimed to detect considerations that may be specific to EOH decisions and are not necessarily encompassed within existing GRADE EtD framework perspectives. Although we identified information to augment the GRADE EtD framework and guidance, our results do not support the addition of any new decision criteria to the GRADE EtD for health system and public health decisions to improve its suitability for EOH decision scenarios. The themes described above (accounting for broad constituencies, consent, timing, the precautionary principle, and social and environmental justice) represent concepts that are accommodated within the GRADE EtD framework criteria but may be more explicitly addressed via tailoring of the framework.

The GRADE EtD framework provides latitude for decision-makers to determine the level of detail of the considerations that they deem relevant to each of the assessment criteria, provided that the process is transparently reported. For example, it may be helpful for an EOH EtD to emphasize certainty of values and certainty of acceptability as considerations where values and acceptability are expected to be key drivers of a recommendation. Disaggregating these considerations could be useful to describe uncertainty about or variability in stakeholder preferences when making a decision that will affect a broad or undefined population. Finally, the cross-cutting themes – time, consent, and the precautionary principle – may inform tailoring of the wording of guidance for assessment criteria and implementation considerations to make the GRADE EtD more applicable in EOH settings. In particular, decision-makers may find it useful to incorporate the precautionary principle as an explicit consideration when assessing the desirable effects, undesirable effects, and balance of effects of the options under consideration.

### 4.5. Unanswered questions and future directions

Feedback from the Delphi panel indicates the importance of additional guidance for GRADE EtD operationalization to facilitate uptake among EOH decision-makers, especially those who are less familiar with the GRADE approach. This could include principles for ethical or equitable decision-making, definitions of terminology used within the GRADE EtD framework, and additional options for signaling questions that are relevant to specific contexts. Subsequent research can include the development and pilot testing of framework implementation guidance with relevant stakeholders.

As mentioned above, the research team and Delphi panel engaged in this study may not be representative of all perspectives in EOH decision-making. Additional user testing of the framework in both hypothetical and real-world scenarios will inform further conclusions about the applicability of the GRADE EOH EtD framework in different contexts.

## 5. Conclusions

The proposed GRADE EtD for EOH resulting from this study is similar to its foundational framework, the GRADE EtD for health system and public health decisions. Our work thus far has served to validate and extend the constructs of the GRADE EtD to a new perspective. Findings of the Delphi process also indicate that the literature is reasonably comprehensive of EOH decision considerations, though further user testing of a proposed framework will provide additional insight. This work represents another step towards development of an EtD to support transparent, explainable decision-making in EOH. Future work will pilot test the proposed framework with a hypothetical decision scenario, present a final version, and provide guidance for its application.

## Supplementary Material

SI

## Figures and Tables

**Table 1:** Example round 1 coding decisions.

Identified Decision Consideration	Code(s) Applied	Rationale

Do any of the proposed interventions feature courses of action that may be against the law?	Feasibility	Maps to detailed judgement, “Are there important legal or bureaucratic or ethical constraints that make it difficult or impossible to cover the intervention?”
People’s reaction: Is there public reaction due to risk, odor, noise, etc.?	Acceptability	Maps to detailed judgments, “Are there key stakeholders that would not accept the distribution of the benefits, harms and costs?” and, “Are there key stakeholders that would not accept the costs or undesirable effects in the short term for desirable effects (benefits) in the future?”
Contaminant source: Hazard inventory and information on severity of hazards	Problem Undesirable Effects	Maps to detailed judgments, “Are the consequences of the problem serious?” (Problem) and main criteria judgment, “How substantial are the undesirable anticipated effects?” (Undesirable Effects)

**Table 2:** Glossary of terms.

Term	Definition

Assessment criteria/on*	Decision factors that are intended to facilitate comparison of alternatives (Problem, Desirable effects, Undesirable effects, Certainty of evidence, Values, Balance of effects, Resources required, Cost effectiveness, Equity, Acceptability, Feasibility)
Decision criteria/on	Factors that should be considered when making a decision or a recommendation
Decision framework	A structured presentation of factors and information to consider when making a decision or a recommendation
Detailed judgement*	An optional prompt intended to facilitate discussion and clarify the information used to inform the assessment criteria judgments
Item	A prompt or signaling question within an EOH decision framework; corresponds to the GRADE EtD framework detailed judgment in level of granularity
Scope and context criteria/on*	Background information, such as the setting and stakeholders, that describes the circumstances under which a decision will be made.

* Language specific to the GRADE EtD framework.

**Table 3:** Characteristics of included EOH decision-making frameworks.

Author, Year	Framework Title	Development Organization	Topic	Reported Decision Criteria

Adar, et al., 2020	Multi-criteria decision-making (MCDM) prioritization of the treatment and disposal methods of wastes	Academic	Sanitation	• Technology • Cost • Environmental • Social/ergonomics
Anonymous, 2014	EPA Framework for Human Health Risk Assessment to Inform Decision Making	Government	Unspecified	• Laws and regulatory requirements • Economic analyses • Sustainability • Technological considerations • Political considerations • Public and social considerations
Anonymous, 2018	Health Quality Ontario Health Technology Assessment (HTA) Decision Determinants Framework	Government	Public health	• Overall clinical benefit • Consistency with expected societal and ethical values • Value for money • Feasibility of adoption into the health system
Anonymous, 2019	Scottish Intercollegiate Guidelines Network (SIGN) guideline methodology	Government	Public health	• Is this question a priority? • How sure are we that any given option will work? • Balancing benefits and harms • How do patients value the different outcomes? • Equity • Costs and benefits
Anonymous1, 2020	Procedures for the CADTH pan-Canadian Oncology Drug Review	Government	Public health	• Overall clinical benefit • Alignment with patient values • Cost effectiveness • Feasibility of adoption into health systems
Anonymous2, 2020	WHO Evidence to Decision (EtD) table	Government	Public health	• Policy importance • Desirable effects • Undesirable effects • Evidence certainty • Balance of effects • Generalizability • Equity • Acceptability • Resources required • Feasibility • Sustainability
Anonymous, unknown year	CDC ACIP Evidence to Recommendations Framework	Government	Public health	• Problem • Benefits & harms • Values • Acceptability • Resource use • Equity • Feasibility of adoption into health systems
Bernstein, 2015	California Natural Resources Agency Decision Framework for Platform Decommissioning in California	Government	Water management	• Legal/regulatory • Environmental/ecological • Feasibility/cost • Liability • Cost
Burger and Gochfeld, 2020	Department of Energy Consortium for Risk Evaluation with Stakeholder Participation (CRESP) template of information needs for decision-making about delaying remediation on contaminated lands to protect human health	Government	Site remediation	• Management, planning, and implementation • Source term, pathways, and methods of exposure • Risks and receptors • External drivers
Cappuyns, 2016	Sustainable Remediation Forum — United Kingdom (SuRF-UK)	NGO	Site remediation	• Human health and safety • Neighbourhood and locality • Communities & community involvement • Uncertainty & evidence
Carande-Kulis, 2012	CDC Guidelines and Recommendations Primer	Government	Public health	• Quality of the evidence • Benefits vs. harms • Values and preferences of the target audience • Resources
Deveau, et al., 2015	Potential sources of variability in science and policy decisions taken during the establishment of occupational exposure limits (OELs)	Academic	Workplace	• Risk science decisions • Risk policy decisions
Dorman, et al., 2014	National Academies Framework to Guide Selection of Chemical Alternatives	NGO	Chemical alternatives assessment	• Physiochemical properties • Life cycle thinking
Felknor, et al., 2019	NIOSH BNI method	Government	Workplace	• Burden • Need • Impact
Garfi and Ferrer-Marti, 2011	General criteria for water and basic sanitation projects	Academic	Water management / Sanitation	• Technical • Social • Economic • Environmental
González-Lorenzo, et al., 2015	Proposed conceptual framework to support vaccine adoption and coverage decisions in a health system	Academic	Public health	• Vaccine characteristics • Impact of immunization program • Values and preferences • Resource use • Equity • Feasibility
He and Hung, 2012	Groupe de Recherche en Économie et Développement International (GREDI) Vehicle Emissions Policymaking Criteria	Academic	Emissions	• Cost of implementation • Effectiveness • Effect time • Political or public acceptability • Administer-ability • Degree of deviations from the existing system
Kayman and Logar, 2016	Framework for Training Public Health Practitioners in Crisis Decision-Making	Academic	Disaster/emergency	• Ethical considerations • Political considerations • Logistical considerations
Krahn, et al., 2018	Ontario Decision Framework	Government	Public health	• Contextual factors • Health system feasibility • Benefits and harms • Economics • Patient-centered care
Malekpour, et al., 2013	Sanitation options evaluation criteria and their indicators	Academic	Sanitation	• Exposure to health hazards • Accessibility • Reliability
Malloy, et al., 2013	Alternatives analysis methodology	Academic	Chemical alternatives assessment	• Physical chemical hazards • Human health impact • Ecological impacts • Environmental impacts • Technical feasibility • Economic feasibility
Marazzi, et al., 2020	Earthwatch Institute (Europe) MCDA approach to consumer-based actions to reduce plastic pollution in rivers	NGO	Water management	• Feasibility • Economic impacts • Environmental impact • Other environmental unintended consequences • Potential scale of change • Evidence of impact
Mitchell, et al., 2013	United States Army Corps of Engineers Framework for Exposure-Based Chemical Prioritization	Government	Chemical alternatives assessment	• Chemical properties • Life cycle properties
Moermond, et al., 2012	RIVM Revised Annex XIII of REACH	NGO	Chemical alternatives assessment	• Persistence • Bioaccumulation • Toxicity
Morley, et al., 2017	NIOSH Proposed Ethical Framework for Decision-making about Employee Monitoring	Government	Workplace	• Justification • Optimization • Minimization of harm • Ethical values
Mullen, et al., 2020	Decision criteria used in PHEIC determinations	Academic	Disaster/emergency	• Constitutes an extraordinary event • Public health risk to other states via international spread • Requires a coordinated international response • Sustained community transmission • Gaps in knowledge due to novel agent or limited response experience • Impending mass gathering • Threat to eradication • Complex response settings
Naman and Gibson, 2015	Analysis of the Decision-Making Process for Water and Sewer Services in North Carolina	Academic	Water management / sanitation	• Financing • Government support • Existing infrastructure • Community engagement • Public health
Pang, et al., 2017	Harmful algal bloom management framework	Academic	Water management	• Human health • Environmental impact • Social impact • Technical feasibility
Perez, et al., 2017	REACH-modified GreenSuite	Industry	Chemical alternatives assessment	• Ecological • Health • Safety
Persson, 2016	Core ideas behind the precautionary principle	Academic	Unspecified	• Value of human health and the environment • Irreversibility • False positives versus false negatives
Ramos, 2018	Human Rights-Based Approach to Farmworker Health	Academic	Workplace	• Availability • Accessibility • Acceptability • Quality
Rehfuess, et al., 2019	WHO-INTEGRATE evidence to decision framework	Government	Public health	• Balance of health benefits and harms • Human rights and sociocultural acceptability • Health equity, equality, and nondiscrimination • Societal implications • Financial and economic considerations • Feasibility and health system considerations • Quality of evidence
Resnik, et al., 2018	National Institute of Environmental Health Sciences (NIEHS) Accountability for reasonableness	Government	Unspecified	• Publicity • Relevancy • Revisability • Enforceability
Sampietro-Colom, et al., 2015	AdHopHTA	NGO	Public health	• Health technology and technical characteristics • Health problem and current use of the technology • Clinical effectiveness • Safety aspects • Ethical, organizational, social, and legal aspects • Cost and economic evaluation
Siu, et al., 2021	US Preventive Services Task Force (USPSTF) Procedure Manual	Government	Public health	• Detection • Benefits - evidence • Benefits - linkage coherence • Benefits - magnitude • Harms - evidence • Harms - linkage coherence • Harms - magnitude • Overall certainty • Magnitude of net benefit
Song and Kim, 2021	Ethical evaluation of community water fluoridation	Academic	Water management	• Effectiveness • Proportionality • Necessity/least infringement • Public justification
Williams, et al., 2020	EPA Regional Environmental Science and Sustainability Research Program (RESES) Dredged Material Decision Tool	Government	Water management	• Biophysical environment • Economy • Social • Governance • Built environment
Woods, et al., 2016	Decision support for risk prioritisation of environmental health hazards in a UK city (funded by Public Health England)	Government	Unspecified	• Mortality • Morbidity • Robust evidence • Wellbeing • Sustainability of intervention • Level of regulation

**Table 4:** Contingency table of discovered EOH decision factors coded to each GRADE EtD criterion by framework characteristics.

Framework Characteristics (N = total frameworks, n = total detailed judgements)	Detailed Judgements (frequency / % of row total)										
	Problem	Desirabl effects	Undesirable effects	Certainty of evidence	Values	Balance of effects	Resources required	Cost effectiveness	Equity	Acceptability	Feasibility

***Development organization***											
*Government (N=18, n=275)*	31 / 6.7	67 / 14.6	58 / 12.6	27 / 5.9	19 / 4.1	27 / 5.9	43 / 9.3	15 / 3.3	19 / 4.1	54 / 11.7	100 / 21.7
*NGO (N=5, n=47)*	10 / 12.8	13 / 16.7	20 / 25.6	3 / 3.8	4 / 5.1	3 / 3.8	5 / 6.4	3 / 3.8	1 / 1.3	10 / 12.8	6 / 7.7
*Industry (N=1, n=35)*	35 / 50	0 / 0	35 / 50	0 / 0	0 / 0	0 / 0	0 / 0	0 / 0	0 / 0	0 / 0	0 / 0
*Academic (N=14, n=241)*	80 / 19	24 / 5.7	93 / 22.1	14 / 3.3	31 / 7.4	11 / 2.6	36 / 8.6	14 / 3.3	12 / 2.9	38 / 9	68 / 16.2
***Topic***											
*Site remediation (N=2, n=72)*	4 / 3.2	7 / 5.6	17 / 13.5	6 / 4.8	6 / 4.8	0 / 0	18 / 14.3	2 / 1.6	5 / 3.9	19 / 15.1	42 / 33.3
*Water management (N=7, n=133)*	17 / 6.9	49 / 20	40 / 16.3	5 / 2	12 / 4.9	19 / 7.8	16 / 6.5	7 / 2.9	5 / 2	25 / 10	50 / 20
*Sanitation (N=4, n=60)*	6 / 5.6	6 / 5.6	20 / 18.7	4 / 3.7	7 / 6.5	1 / 0.9	12 / 11.2	4 / 3.7	3 / 2.8	14 / 13.1	30 / 28
*Workplace exposure (N=4, n=35)*	2 / 8.3	2 / 8.3	2 / 8.3	3 / 12.5	2 / 8.3	2 / 8.3	0 / 0	0 / 0	1 / 4.2	8 / 33.3	2 / 8.3
*Emissions (N=1, n=6)*	0 / 0	2 / 22.2	0 / 0	0 / 0	0 / 0	0 / 0	1 / 11.1	1 / 11.1	0 / 0	3 / 33.3	2 / 22.2
*Chemical alternatives identification (N=5, n=113)*	99 / 41.6	1 / 0.4	104 / 43.7	0 / 0	8 / 3.4	2 / 0.8	11 / 4.6	2 / 0.8	0 / 0	3 / 1.3	8 / 3.4
*Public health (N=11, n=132)*	19 / 9.9	24 / 12.6	16 / 8.4	19 / 9.9	12 / 6.3	9 / 4.7	19 / 9.9	13 / 6.8	15 / 7.9	20 / 10.5	25 / 13.1
*Emergency / Disaster (N=2, n=23)*	5 / 12.8	5 / 12.8	1 / 2.6	3 / 7.7	4 / 10	4 / 10	3 / 7.7	2 / 5.1	2 / 5.1	5 / 12.8	5 / 12.8
*Unspecified (N=4, n=24)*	4 / 8	8 / 16	6 / 12	4 / 8	3 / 6	4 / 8	4 / 8	1 12	1 / 2	5 / 10	10 / 20

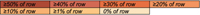

**Table 5:** Summary of GRADE EtD criterion codes and unique codes applied to discovered EOH decision factors.

GRADE EtD Criteria (codes)	Total Discovered Items (frequency / % of total)	Redundant (frequency / % of row)	Adds to Guidance (frequency / % of row)	Adds to Scope/Context (frequency / % of row)	Adds to Detailed Judgements (frequency / % of row)
	*Total = 560* *	*n = 930* *	*n = 2*	*n = 40*	*n = 62*

*Problem*	156 / 27.9	115 / 73.7	2 / 1.3	14 / 8.9	25 / 16
*Desirable effects*	104 / 18.6	99 / 95.2	0 / 0	0 / 0	5 / 4.8
*Undesirable effects*	206 / 36.8	204 / 99	0 / 0	0 / 0	2 / 1
*Certainty of evidence*	44 / 7.9	44 / 100	0 / 0	0 / 0	0 / 0
*Values*	54 / 9.6	48 / 88.9	0 / 0	6 / 11.1	0 / 0
*Balance of effects*	41 / 7.3	41 / 100	0 / 0	0 / 0	0 / 0
*Resources required*	84 / 15.0	59 / 70.2	0 / 0	6 / 7.1	19 / 22.6
*Cost effectiveness*	32 / 5.7	27 / 84.4	0 / 0	2 / 6.3	3 / 9.4
*Equity*	32 / 5.7	27 / 84.4	0 / 0	1 / 3.1	4 / 12.5
*Acceptability*	102 / 18.2	94 / 93.1	0 / 0	6 / 5.9	2 / 2
*Feasibility*	174 / 31.1	171 / 98.3	0 / 0	3 / 1.7	0 / 0

* Multiple codes were applied to some detailed judgements; these totals include each time a detailed judgement was coded to a criterion and thus, count some detailed judgements more than once.

**Table 6:** Delphi panel demographic characteristics.

Demographic Category	Round 1 (n = 20) n (%)	Round 2 (n = 19) n (%)

*Gender*		
Female	10 (50)	9 (47)
Male	10 (50)	10 (53)
*Geographical region*		
Africa	1 (5 %)	1 (5 %)
Asia	3 (15 %)	2 (11 %)
Australia	2 (10 %)	2 (11 %)
Europe	4 (20 %)	4 (21 %)
North America	9 (45 %)	9 (47 %)
South America	1 (5 %)	1 (5 %)
*Setting*		
Government or regulatory agency	10 (50 %)	10 (53)
Academia or research	10 (50 %)	9 (47)
Area of expertise*		
Cancer	1	1
Environmental health	10	10
Food safety or nutrition	1	1
Occupational health	5	4
Risk assessment or management	1	1
Other	4	4

* Multiple areas of expertise may be attributed to a single participant.

**Table 7:** Summary of decisions based on two rounds of Delphi panel ratings.

ROUND 1		ROUND 2	
Content Removed (n = 27)	Content for Re-Rating (n = 56)	Content Removed (n = 5)	Proposed Framework (n = 47)

Background questions or judgments (e.g., availability of data, mandate of the decision-maker) Redundant considerations (e. g., multiple criteria for judging the toxicity of an exposure) Implementation considerations (e. g., quality of communication plan) Material that was rated as unhelpful for decision-making because the phrasing was uninterpretable	Re-worded criteria and detailed judgments from the GRADE EtD for health system and public health decisions Certainty of cost-effectiveness Social justice considerations (e. g., issues of intergenerational equity) Acceptability and feasibility considerations related to laws and regulations (e.g., current laws and regulations applying to worker safety)	Community engagement in advocacy or organizing as an indicator of priority, as this factor may or may not be present regardless of priority Risk tolerance as a separate consideration from other values Laws and regulations as a consideration to inform judgments about the acceptability of an intervention Perception of feasibility as a consideration to inform judgments about feasibility	Problem: addition of irreversibility and precedent as considerations Desirable / Undesirable Effects: addition of time span to full effectiveness Resources Required / Certainty of Resources: simplified detailed judgments Cost Effectiveness: consolidated detailed judgments to certainty in analyses Equity: broadening considerations to include social and environmental justice in addition to health equity Acceptability: addition of certainty in estimates of acceptability Feasibility: addition of time span for sustainability and tailoring of types of barriers/enablers to implementation

## Data Availability

Data and code are shared on GitHub
