# Supplementary material for: Development of a framework to structure decision-making in environmental and occupational health: A systematic review and Delphi study
Source: Environ Int. Author manuscript; Available in PMC 2026 Jan 1. (PMC12026343; doi:10.1016/j.envint.2024.109209)
Supplement: SI [file NIHMS2055162-supplement-SI.docx]

**Supplement to “Development of a framework to structure decision-making in environmental and occupational health: a systematic review and Delphi study”**

**Supplement Table of Contents**

Supplement A. Database search strategy 2

Supplement B. Grey literature search strategy 5

Supplement C. Abstraction instrument 9

Supplement D. Documents excluded at full text screening 14

Supplement E. GRADE Evidence to Decision (EtD) framework for health system and public health decisions 34

Supplement E. Unique EOH decision factors organized by related GRADE EtD assessment criteria 37

Supplement F. Round 1 Delphi rating instrument 40

Supplement G. Round 2 Delphi rating instrument 51

# Supplement A. Database search strategy

Search date: September 26, 2021

Literature search performed by: Emily Senerth

Search Strategy:

--------------------------------------------------------------------------------

**Database: Ovid MEDLINE(R) ALL / PubMed(R) <1946 to Present>**

1 exp *Occupational Health/ 25331

2 exp *Occupational Exposure/ 43236

3 exp *Occupational Diseases/ 114368

4 exp *Occupational Medicine/ 17832

5 exp *Environment/ and Public Health/ 4901

6 exp *Environment, Controlled/ 79973

7 exp *Environmental Exposure/ 187600

8 exp *Environmental Health/ 17029

9 exp *Environmental Pollutants/ 261689

10 exp *Environmental Monitoring/ 75753

**11 1-10 627972**

12 exp *Social Control, Formal/ 427891

13 exp *Decision Making/ 100339

14 exp *Decision Support Techniques/ 27699

15 exp *evidence based medicine/ 26741

16 (evidence-based adj2 (analys* or assess*)).ti,ab. 2097

17 (decision* adj2 (determinant* or model* or analysis* or technique* or framework* or tool* or template*)).ti. 5573

18 (decisionmaking or decision-making).ti. 29672

19 (evidence-to-decision or evidence to decision).ti,ab. 2490

20 ((grade or grading) adj2 (evidence or recommendation*)).ti,ab. 8646

21 (strength of evidence or quality of evidence or strength of recommendation*).ti,ab. 22475

22 exp Review Literature as Topic/ 17771

23 exp *Policy Making/ 9685

**24 12-23 628168**

**25 11 and 24 10304**

26 limit 25 to english language 8805

27 limit 26 to yr="2011 - 2021" 3674

--------------------------------------------------------------------------------

**Database: SCOPUS Embase <1996 to Present>**

( INDEXTERMS ( "Occupational Health" )  OR  INDEXTERMS ( "Occupational Exposure" )  OR  INDEXTERMS ( "Occupational Diseases" )  OR  INDEXTERMS ( "Occupational Medicine" )  OR  INDEXTERMS ( environment )  AND  INDEXTERMS ( "Public Health" )  OR  INDEXTERMS ( "Environment, Controlled" )  OR  INDEXTERMS ( "Environmental Exposure" )  OR  INDEXTERMS ( "Environmental Health" )  OR  INDEXTERMS ( "Environmental Pollutants" )  OR  INDEXTERMS ( "Environmental Monitoring" ) )  AND  ( INDEXTERMS ( "Social Control, Formal" )  OR  INDEXTERMS ( "Decision Making" )  OR  INDEXTERMS ( "Decision Support Techniques" )  OR  INDEXTERMS ( "evidence based medicine" )  OR  TITLE-ABS ( evidence-based  W/2  ( analys*  OR  assess* ) )  OR  TITLE ( decision*  W/2  ( determinant*  OR  model*  OR  analysis*  OR  technique*  OR  framework*  OR  tool*  OR  template* ) )  OR  TITLE ( decisionmaking  OR  decision-making )  OR  TITLE-ABS ( evidence-to-decision  OR  "evidence to decision" )  OR  TITLE-ABS ( ( grade  OR  grading )  W/2  ( evidence  OR  recommendation* ) )  OR  TITLE-ABS ( "strength of evidence"  OR  "quality of evidence"  OR  "strength of recommendation*" )  OR  INDEXTERMS ( "Review Literature as Topic" )  OR  INDEXTERMS ( "Policy Making" ) )  AND  ( LIMIT-TO ( PUBYEAR ,  2021 )  OR  LIMIT-TO ( PUBYEAR ,  2020 )  OR  LIMIT-TO ( PUBYEAR ,  2019 )  OR  LIMIT-TO ( PUBYEAR ,  2018 )  OR  LIMIT-TO ( PUBYEAR ,  2017 )  OR  LIMIT-TO ( PUBYEAR ,  2016 )  OR  LIMIT-TO ( PUBYEAR ,  2015 )  OR  LIMIT-TO ( PUBYEAR ,  2014 )  OR  LIMIT-TO ( PUBYEAR ,  2013 )  OR  LIMIT-TO ( PUBYEAR ,  2012 )  OR  LIMIT-TO ( PUBYEAR ,  2011 ) )  AND  ( LIMIT-TO ( LANGUAGE ,  "English" ) )

--------------------------------------------------------------------------------

**Database: Cochrane Library**

[mh "Occupational Health"]

[mh "Occupational Exposure"]

[mh "Occupational Diseases"]

[mh "Occupational Medicine"]

[mh “Environment and Public Health"]

[mh "Environment, Controlled"]

[mh "Environmental Exposure"]

[mh "Environmental Health"]

[mh "Environmental Pollutants"]

[mh "Environmental Monitoring"]

[mh "Social Control, Formal"]

[mh "Decision Making"]

[mh "Decision Support Techniques"]

[mh "evidence based medicine"]

(evidence-based:ti,ab NEAR/2 (analys*:ti,ab OR assess*:ti,ab))

(decision*:ti NEAR/2 (determinant*:ti OR model*:ti OR analysis*:ti OR technique*:ti OR framework*:ti OR tool*:ti OR template*:ti))

(decisionmaking:ti OR decision-making:ti)

(evidence-to-decision:ti,ab OR "evidence to decision":ti,ab)

((grade:ti,ab OR grading:ti,ab) NEAR/2 (evidence:ti,ab OR recommendation*:ti,ab))

("strength of evidence":ti,ab OR "quality of evidence":ti,ab OR ("strength of recommendation”*):ti,ab)

[mh "Review Literature as Topic"]

[mh "Policy Making"]

# Supplement B. Grey literature search strategy

| **Date** | **Organization name & URL** | **Search strategy(s) / words searched** | **# documents retrieved*** | **# documents included** |
| --- | --- | --- | --- | --- |
| 1/3/22 | Canadian Standards Association (CSA)  https://www.csagroup.org/about-csa-group/ | Searched decision [and] framework/model/tool/analysis, decision-making, recommendation(s), guideline(s), methodology | 0 items screened | 0 |
| 1/3/22 | Chartered Institute of Environmental Health (CIEH)  https://www.cieh.org | Searched decision [and] framework/model/tool/analysis, decision-making, recommendation(s), guideline(s), methodology | 1 item screened | 0 |
| 1/3/22 | International Network of Agencies for Health Technology Assessment (INAHTA)  https://www.inahta.org | Searched decision [and] framework/model/tool/analysis, decision-making, recommendation(s), guideline(s), methodology | 3 items screened | 1 |
| 1/3/22 | Guidelines International Network  https://g-i-n.net | Reviewed “resources” tab | 0 items screened | 0 |
| 1/3/22 | International Federation of Environmental Health (IFEH)  https://www.ifeh.org/index.html | Searched decision [and] framework/model/tool/analysis, decision-making, recommendation(s), guideline(s), methodology | 5 items screened | 0 |
| 1/3/22 | Air & Waste Management Association (AWMA)  https://www.awma.org | Searched decision [and] framework/model/tool/analysis, decision-making, recommendation(s), guideline(s), methodology | 0 items screened | 0 |
| 1/3/22 | American Water Works Association  https://www.awwa.org | Reviewed “publications” tab | 0 items screened | 0 |
| 1/3/22 | Children’s Environmental Health Network (CEHN)  https://cehn.org | Searched decision framework, guidelines | 0 items screened | 0 |
| 1/3/22 | Environmental and Occupational Health Sciences Institute (EOHSI)  https://eohsi.rutgers.edu | Searched decision [and] framework/model/tool/analysis, decision-making, recommendation(s), guideline(s), methodology | 1 item screened | 0 |
| 1/3/22 | Health Effects Institute (HEI)  https://www.healtheffects.org | Searched decision [and] framework/tool, decision-making, guideline(s), methodology | 1 item screened | 0 |
| 1/3/22 | US Climate and Health Alliance  https://usclimateandhealthalliance.org | Searched decision [and] framework/tool, decision-making, guideline(s), methodology | 2 items screened | 0 |
| 1/3/22 | Public Health Agency of Canada  https://www.canada.ca/en/public-health.html | Reviewed mandate – unable to restrict search to public health agency website | 0 items screened | 0 |
| 1/3/22 | Health Canada  https://www.canada.ca/en/health-canada.html | Reviewed publications - guidelines | 3 items screened | 0 |
| 1/3/22 | Environment Canada  https://www.canada.ca/en/environment-climate-change.html | Reviewed publications | 1 item screened | 0 |
| 1/3/22 | World Health Organization (WHO) and International Labour Organization  https://www.who.int/publications/who-guidelines | Reviewed guidelines page; searched for guideline [and] methods/methodology | 9 items screened | 2 |
| 1/3/22 | Canadian Agency for Drugs and Technologies in Health (CADTH)  https://www.cadth.ca/about-cadth | Searched decision [and] framework/tool/criteria, decision-making, manual, methodology | 59 items screened | 1 |
| 1/3/22 | Health Quality Ontario  https://www.hqontario.ca | Reviewed “evidence to improve care” page | 5 items screened | 1 |
| 1/3/22 | National Institute for Health and Care Excellence (NICE)  https://www.nice.org.uk/about | Reviewed guidance – guidelines – guideline development process | 1 item screened | 0 |
| 1/3/22 | British Columbia Ministry of Health  https://www2.gov.bc.ca/gov/content/home | Reviewed guidelines – GPAC handbook | 1 item screened | 0 |
| 1/3/22 | Scottish Intercollegiate Guidelines Network (SIGN)  https://www.sign.ac.uk | Reviewed “what we do” - methodology | 3 items screened | 1 |
| 1/3/22 | European Food Safety Authority (EFSA)  https://www.efsa.europa.eu/en | Reviewed about – “how we work” and resources - methodology | 7 items screened | 0 |
| 1/4/22 | ECRI  <https://www.ecri.org> | Searched library for decision [and] framework/tool, decision-making, methodology | 25 items screened | 0 |
| 1/4/22 | Centers for Disease Control and Prevention (CDC)  https://www.cdc.gov/about/ | Searched decision [and] framework/tool, decision-making, methodology | 120 items screened | 2 |
| 1/4/22 | National Institute for Occupational Safety and Health (NIOSH)  https://www.cdc.gov/NIOSH/ | Searched decision [and] framework/tool, decision-making, methodology | 40 items screened | 2 |
| 1/4/22 | Occupational Safety and Health Administration (OSHA)  https://www.osha.gov | Searched decision [and] framework/tool, methodology | 19 items screened | 0 |
| 1/4/22 | Environmental Protection Agency (EPA)  https://www.epa.gov | Searched decision [and] framework/tool, decision-making [and] methodology | 90 items screened | 2 |
| 1/5/22 | Science.gov  https://www.science.gov | Searched decision framework and restricted results to topics “EPA” [or] “decision-making” from 2011 to 2022 | 134 items screened | 1 |
| 1/5/22 | Agency for Healthcare Research and Quality  https://www.ahrq.gov | Searched decision [and] framework | 24 items screened | 1 |
| 1/6/22 | NIH National Institute of Environmental Health Sciences  https://www.niehs.nih.gov | Searched decision [and] framework/tool, decision-making | 41 items screened | 1 |
| 1/6/22 | National Academies of Sciences, Engineering, Medicine  https://www.nationalacademies.org | Searched decision [and] framework/tool | 105 items screened | 1 |
| 1/6/22 | US Preventive Services Taskforce  https://www.uspreventiveservicestaskforce.org/uspstf/ | Searched decision [and] framework | 27 items screened | 0 |
| 1/6/22 | Federal Register  https://www.federalregister.gov | Searched decision [and] framework, and restricted results to topics “environment” [or] “health & public welfare” | 108 items screened | 0 |
| 10/24/24 | European Agency for Safety and Health at Work (EU-OSHA)  https://osha.europa.eu/en | Searched the terms “decision,” “decision-making” and “framework” from website homepage  Reviewed the methods and references from two recent European Guides (“Heat at work” and “Guidance for the safe management of hazardous medicinal products at work”) for related technical documents | 64 items screened | 0 |
| 10/24/24 | Korean Occupational Safety and Health Administration (KOSHA)  https://www.kosha.or.kr/english/index.do | Searched the terms “decision,” “decision-making” and “framework” on landing pages for Publications and Legislation  Reviewed recent documents in the “Resources” and “Publications” sections | 20 items screened | 0 |
| 10/24/24 | International Commission on Occupational Health (ICOH)  https://www.icohweb.org/site/homepage.asp | Searched the terms “decision,” “decision-making” and “framework” from website homepage  Reviewed the “core documents” area of the website, containing organizational documents  Reviewed the methods and references from most recent guideline document (“Ergonomics Guidelines”) for related technical documents | 48 items screened | 0 |
| 10/24/24 | Canadian Centre for Occupational Health and Safety (CCOHS)  https://www.ccohs.ca | Searched the terms “decision,” “decision-making” and “framework” from website homepage  Specific management documents only available with purchase | 30 items screened | 0 |
| TOTAL |  |  | 997 items screened | 16 items moved to extraction |

* *All search results were reviewed for relevance by 1 reviewer*

# Supplement C. Abstraction instrument

1. Reviewer's initials *

2. Last name of primary author *

3. Publication year *

4. Journal title *

5. Document is in English *

*Mark only one oval.*

Yes No

**Study characteristics**

6. Study presents a decision-making framework *

*Mark only one oval.*

Yes No

7. Framework is used to formulate environmental or occupational health (EOH) threshold or acceptable exposure level *

*Mark only one oval.*

Yes No

8. Framework is used to come to an agreement on whether or not to recommend a public health/EOH intervention *

*Mark only one oval.*

Yes No

9. Framework is used for prioritization of public health/EOH interventions or hazards *

*Mark only one oval.*

Yes No

10. Framework is used to inform public health/EOH policy *

*Mark only one oval.*

Yes No

**Study design**

11. Study design *

*Mark only one oval.*

Case study

Systematic review of decision-making frameworks

Editorial/Commentary

Other:

**The Framework (General Overview)**

12. Title of the framework * (Or "no title")

13. Primary developer of the framework *

14. Primary user of the framework *

15. Please list all topics (e.g., air quality, noise, etc.) that the framework is being applied to *

16. Country in which the framework is being applied (List all). *

17. Country in which framework was developed (List all) *

18. Audience that the framework is intending to inform *

*Check all that apply.*

Policymakers

Stakeholders

Researchers

General population

Other:

**The Framework (Specific Components)**

19. How many domains does the framework have?

20. What is the name of the first domain

21. List all questions within the first domain

22. Framework has an additional domain

*Mark only one oval.*

Yes No

**Second Domain**

23. What is the name of the next domain

24. List all questions within the second domain

25. Framework has an additional domain

*Mark only one oval.*

Yes No

**Third Domain**

26. What is the name of the next domain

27. List all questions within the third domain

28. Framework has an additional domain

*Mark only one oval.*

Yes No

**Fourth Domain**

29. What is the name of the next domain

30. List all questions within the fourth domain

31. Framework has an additional domain

*Mark only one oval.*

Yes No

**Fifth Domain**

32. What is the name of the next domain

33. List all questions within the fifth domain

34. Framework has an additional domain

*Mark only one oval.*

Yes No

**Sixth Domain**

35. What is the name of the next domain

36. List all questions within the sixth domain

37. Framework has an additional domain

*Mark only one oval.*

Yes No

**Seventh Domain**

38. What is the name of the next domain

39. List all questions within the seventh domain

40. Framework has an additional domain

*Mark only one oval.*

Yes No

**Remaining Domains**

41. List all remaining domains and questions

**Decision Criteria**

42. Were criteria used to determine the overall decision about a recommendation, threshold, or policy?

*Mark only one oval.*

Yes No

43. Describe the criteria used to determine the overall decision.

*Copy and paste from text*

44. How were the criteria used to inform the overall decision?

*Mark only one oval.*

Weights

Judgment

Other:

**Quality of the Framework**

45. Does the framework advance research on the topic?

*If yes, copy and paste from text. If not, answer "No" or "Not discussed"*

46. Does the framework advance research on decision-making frameworks?

*If yes, copy and paste from text. If not, answer "No" or "Not discussed"*

47. Is there research supporting the overall decision of the framework?

*If yes, copy and paste from text. If not, answer "No" or "Not discussed"*

48. How will the overall decision be considered at the policy level?

*If yes, copy and paste from text. If not, answer "No" or "Not discussed"*

49. What is the weight that this decision/the research evidence carry at the national level?

*If yes, copy and paste from text. If not, answer "No" or "Not discussed"*

**Context of the Framework**

50. Does the framework take into consideration whether or not the issue is polarizing?

*If yes, copy and paste from text. If not, answer "No" or "Not discussed"*

51. Does the framework account for the fact that care options may vary in their salience to the patients and the public?

*If yes, copy and paste from text. If not, answer "No" or "Not discussed"*

52. Does the framework speak to whether the political systems are more or less amenable to the issue?

*If yes, copy and paste from text. If not, answer "No" or "Not discussed"*

**Other Considerations**

53. Funding sources disclosed in the study

54. Study limitations

55. Review references from the study. Are any relevant to this study and should be pulled for further review

# Supplement D. Documents excluded at full text screening

| **Study** | **Title** | **Exclusion Reason** |
| --- | --- | --- |
| Abdelzaher 2013 | An alternative approach to water regulations for public health protection at bathing beaches. | No public health/EOH decision-framework |
| Acir 2018 | Endocrine-disrupting metabolites of alkylphenol ethoxylates - A critical review of analytical methods, environmental occurrences, toxicity, and regulation. | No public health/EOH decision-framework |
| Agerstrand 2017 | An academic researcher's guide to increased impact on regulatory assessment of chemicals. | No public health/EOH decision-framework |
| Ahlers 2019 | Environmental hazard and risk assessment of thiochemicals. Application of integrated testing and intelligent assessment strategies (ITS) to fulfil the REACH requirements for aquatic toxicity. | Hazard/risk identification framework |
| Akhtar 2013 | GLIMPSE: a rapid decision framework for energy and environmental policy. | No public health/EOH decision-framework |
| Aliani 2016 | Determining an appropriate method for the purpose of land allocation for ecotourism development (case study: Taleghan County, Iran) | No public health/EOH decision-framework |
| Allen 2019 | Collaborative Workshops for Community Meaning-Making and Data Analyses: How Focus Groups Strengthen Data by Enhancing Understanding and Promoting Use. | No public health/EOH decision-framework |
| Alves 2012 | U.S. EPA authority to use cumulative risk assessments in environmental decision-making. | No public health/EOH decision-framework |
| Andersen 2019 | Systematic literature review on the effects of occupational safety and health (OSH) interventions at the workplace. | Evidence appraisal framework |
| Anonymous 2021 | Clinical Guidance to Optimize Work Participation After Injury or Illness: Using the Evidence to Guide Physical Therapist Practice. | No public health/EOH decision-framework |
| Atwood 2019 | New perspectives for cancer hazard evaluation by the report on carcinogens: A case study using read-across methods in the evaluation of haloacetic acids found as water disinfection by-products | Hazard/risk identification framework |
| Austin 2012 | Policy, practice and decision making for zoonotic disease management: water and Cryptosporidium. | No public health/EOH decision-framework |
| Banerjee 2014 | Bayesian hierarchical framework for occupational hygiene decision making. | No public health/EOH decision-framework |
| Bartonova 2012 | How can scientists bring research to use: the HENVINET experience. | No public health/EOH decision-framework |
| Bartonova 2012 | How can scientists bring research to use: The HENVINET experience | No public health/EOH decision-framework |
| Bas 2014 | A framework for child safety and health management by analogy to occupational safety and health management | No public health/EOH decision-framework |
| Bates 2014 | The value of information for managing contaminated sediments. | No public health/EOH decision-framework |
| Bell 2016 | Bread and roses: A gender perspective on environmental justice and public health | No public health/EOH decision-framework |
| Bennett 2018 | Drumming-associated anthrax incidents: Exposures to low levels of indoor environmental contamination | No public health/EOH decision-framework |
| Bevan 2017 | Setting evidence-based occupational exposure limits for manganese. | Hazard/risk identification framework |
| Birnbaum 2012 | Consortium-based science: The NIEHS's multipronged, collaborative approach to assessing the health effects of Bisphenol A | No public health/EOH decision-framework |
| Blumensaat 2012 | Water quality-based assessment of urban drainage impacts in Europe - where do we stand today?. | No public health/EOH decision-framework |
| Bogen 2014 | Unveiling variability and uncertainty for better science and decisions on cancer risks from environmental chemicals. | No public health/EOH decision-framework |
| Booth 2017 | Modeling aesthetics to support an ecosystem services approach for natural resource management decision making. | No public health/EOH decision-framework |
| Brauer 2013 | Just sustainability? Sustainability and social justice in professional codes of ethics for engineers. | No public health/EOH decision-framework |
| Britton 2021 | Accelerating sustainable ocean policy: The dynamics of multiple stakeholder priorities and actions for oceans and human health | No public health/EOH decision-framework |
| Brown 2011 | Science versus policy in establishing equitable Agent Orange disability compensation policy. | No public health/EOH decision-framework |
| Brown 2018 | Evolution of the United States Energy System and Related Emissions under Varying Social and Technological Development Paradigms: Plausible Scenarios for Use in Robust Decision Making. | Hazard/risk identification framework |
| Browne 2017 | The case for environment in all policies: Lessons from the health in all policies approach in public health | No public health/EOH decision-framework |
| Bures 2012 | A proposal for a computer-based framework of support for public health in the management of biological incidents: the Czech Republic experience. | No public health/EOH decision-framework |
| Caquilpan 2019 | Advantages and challenges of the implementation of a low-cost particulate matter monitoring system as a decision-making tool. | No public health/EOH decision-framework |
| Carriger 2012 | Influence diagrams as decision-making tools for pesticide risk management. | No public health/EOH decision-framework |
| Carter 2012 | Environmental planning and management in an age of uncertainty: the case of the Water Framework Directive. | No public health/EOH decision-framework |
| Ceccato 2018 | Data and tools to integrate climate and environmental information into public health | No public health/EOH decision-framework |
| Chang 2019 | Policy changes for preventing and recognizing overwork-related cardiovascular diseases in Taiwan: An overview. | No public health/EOH decision-framework |
| Chari 2018 | Expanding the Paradigm of Occupational Safety and Health: A New Framework for Worker Well-Being. | No public health/EOH decision-framework |
| Chen 2012 | Optimizing decision making for late-phase recovery one year after the Fukushima nuclear accident. | No public health/EOH decision-framework |
| Chen 2018 | Decision-Making and Environmental Implications under Cap-and-Trade and Take-Back Regulations. | No public health/EOH decision-framework |
| Chen 2018 | Processing Technology Selection for Municipal Sewage Treatment Based on a Multi-Objective Decision Model under Uncertainty. | No public health/EOH decision-framework |
| Cherednichenko 2020 | Formal Modeling of Decision-Making Processes Under Transboundary Emergency Conditions | No public health/EOH decision-framework |
| Choi 2021 | Mainstreaming of health equity in infectious disease control policy during the covid-19 pandemic era | No public health/EOH decision-framework |
| Chughtai 2013 | Availability, consistency and evidence-base of policies and guidelines on the use of mask and respirator to protect hospital health care workers: a global analysis. | No public health/EOH decision-framework |
| Chughtai 2020 | Policies on the use of respiratory protection for hospital health workers to protect from coronavirus disease (COVID-19). | No public health/EOH decision-framework |
| Clark 2018 | WHO Environmental Noise Guidelines for the European Region: A Systematic Review on Environmental Noise and Quality of Life, Wellbeing and Mental Health. | No public health/EOH decision-framework |
| Collins 2011 | Risk-based targeting: identifying disproportionalities in the sources and effects of industrial pollution. | No public health/EOH decision-framework |
| Colombo 2018 | Decision-making in humanitarian crises: politics, and not only evidence, is the problem. | No public health/EOH decision-framework |
| Comber 2013 | Development of a chemical source apportionment decision support framework for catchment management. | No public health/EOH decision-framework |
| Costa-Souza 2018 | A socio-historical approach to policy analysis: The case of the Brazilian workers food policy | No public health/EOH decision-framework |
| Couch 2011 | Community stress, psychosocial hazards, and EPA decision-making in communities impacted by chronic technological disasters. | No public health/EOH decision-framework |
| Culin 2018 | Brominated flame retardants: Recommendation for different listing under the Hong Kong Convention. | No public health/EOH decision-framework |
| Currie 2018 | The application of system dynamics modelling to environmental health decision-making and policy - a scoping review. | No public health/EOH decision-framework |
| Dainiak 2011 | Literature review and global consensus on management of acute radiation syndrome affecting nonhematopoietic organ systems. | No public health/EOH decision-framework |
| Dainiak 2011 | First global consensus for evidence-based management of the hematopoietic syndrome resulting from exposure to ionizing radiation. | No public health/EOH decision-framework |
| Davila 2011 | Definition and insertion of the GSPC in the political context of Mexico. | No public health/EOH decision-framework |
| Declet-Barreto 2020 | Hazardous air pollutant emissions implications under 2018 guidance on U.S. Clean Air Act requirements for major sources. | No public health/EOH decision-framework |
| Dellarco 2017 | Using exposure bands for rapid decision making in the RISK21 tiered exposure assessment. | Hazard/risk identification framework |
| Delpla 2014 | A decision support system for drinking water production integrating health risks assessment. | Hazard/risk identification framework |
| Devos 2014 | Towards a more open debate about values in decision-making on agricultural biotechnology. | No public health/EOH decision-framework |
| Ding 2011 | Occupational exposure limits in Europe and Asia--continued divergence or global harmonization?. | No public health/EOH decision-framework |
| Driver 2019 | Utilization of the Maryland Environmental Justice Screening Tool: A Bladensburg, Maryland Case Study. | No public health/EOH decision-framework |
| Duhamel 2013 | Creating a fall protection plan and a safer workplace. | No public health/EOH decision-framework |
| Ellickson 2011 | Cumulative risk assessment and environmental equity in air permitting: interpretation, methods, community participation and implementation of a unique statute. | Hazard/risk identification framework |
| Elwell 2018 | Using people's perceptions of ecosystem services to guide modeling and management efforts | No public health/EOH decision-framework |
| Fann 2011 | Maximizing health benefits and minimizing inequality: incorporating local-scale data in the design and evaluation of air quality policies. | No public health/EOH decision-framework |
| Fenech 2014 | Development of a decision-support tool for identifying the most suitable approach to achieve nitrate source determination. | No public health/EOH decision-framework |
| Fojcikova 2019 | ESTE-DECISION SUPPORT SYSTEM FOR NUCLEAR AND RADIOLOGICAL ACCIDENTS. | No public health/EOH decision-framework |
| FoodandDrugAdministration 2012 | International Conference on Harmonisation; final recommendation for the revision of the permitted daily exposure for the solvent cumene according to the maintenance procedures for the guidance Q3C Impurities: Residual Solvents; availability. Notice. | No public health/EOH decision-framework |
| Ford 2017 | A Critique of Risk Disclosure as the Solution for Minimizing Toxic Exposures in Pregnancy. | No public health/EOH decision-framework |
| Friesen 2016 | Combining Decision Rules from Classification Tree Models and Expert Assessment to Estimate Occupational Exposure to Diesel Exhaust for a Case-Control Study | Evidence appraisal framework |
| Furlong 2011 | Governance and sustainability at a municipal scale: the challenge of water conservation. | No public health/EOH decision-framework |
| Ganesh 2018 | Climate Change, Public Health, and Policy: A California Case Study | No public health/EOH decision-framework |
| Gangwal 2012 | Incorporating exposure information into the toxicological prioritization index decision support framework. | Hazard/risk identification framework |
| Gao 2017 | Indicators' role: How do they influence Strategic Environmental Assessment and Sustainable Planning - The Chinese experience. | No public health/EOH decision-framework |
| Garcia-Alonso 2014 | Protection goals in environmental risk assessment: a practical approach. | No public health/EOH decision-framework |
| Garriga 2015 | Improved monitoring framework for local planning in the water, sanitation and hygiene sector: From data to decision-making. | No public health/EOH decision-framework |
| Gasperini 2017 | Public Policy and the Next Generation of Farmers, Ranchers, Producers, and Agribusiness Leaders. | No public health/EOH decision-framework |
| Genereux 2019 | From Science to Policy and Practice: A Critical Assessment of Knowledge Management before, during, and after Environmental Public Health Disasters. | No public health/EOH decision-framework |
| Gibson 2011 | A burden of disease approach to prioritizing environmental policy initiatives: a case study in the Middle East. | No public health/EOH decision-framework |
| Ginsberg 2019 | New Toxicology Tools and the Emerging Paradigm Shift in Environmental Health Decision-Making. | No public health/EOH decision-framework |
| Giubilato 2014 | A risk-based methodology for ranking environmental chemical stressors at the regional scale. | No public health/EOH decision-framework |
| Gomez 2019 | Literature Review of Policy Implications From Findings of the Center for Work, Health, and Well-being. | No public health/EOH decision-framework |
| Gordon 2011 | Structuring expert input for a knowledge-based approach to watershed condition assessment for the Northwest Forest Plan, USA | No public health/EOH decision-framework |
| Greer 2018 | Labour politics as public health: how the politics of industrial relations and workplace regulation affect health. | No public health/EOH decision-framework |
| Gross 2017 | Regulating toxic chemicals for public and environmental health. | No public health/EOH decision-framework |
| Guski 2017 | WHO Environmental Noise Guidelines for the European Region: A Systematic Review on Environmental Noise and Annoyance. | No public health/EOH decision-framework |
| Gwinn 2017 | Chemical Risk Assessment: Traditional vs Public Health Perspectives. | Hazard/risk identification framework |
| Hall 2017 | New approach to weight-of-evidence assessment of ecotoxicological effects in regulatory decision-making. | Hazard/risk identification framework |
| Hall 2017 | Supporting the eu response to environmental emergencies: European multiple environmental threats emergency network | Hazard/risk identification framework |
| He 2021 | A Risk and Decision Analysis Framework to Evaluate Future PM2.5 Risk: A Case Study in Los Angeles-Long Beach Metro Area. | No public health/EOH decision-framework |
| Hegmann 2013 | ACOEM practice guidelines: elbow disorders. | No public health/EOH decision-framework |
| Henderson 2012 | A data-driven approach to setting trigger temperatures for heat health emergencies. | No public health/EOH decision-framework |
| Hengpraprom 2011 | Developing tools for health impact assessment in environmental impact assessment in Thailand. | Hazard/risk identification framework |
| Hinchcliff 2017 | The enhanced knowledge translation and exchange framework for road safety: A brief report on its development and potential impacts | No public health/EOH decision-framework |
| Ho 2020 | Comparison of freshwater monitoring approaches: strengths, opportunities, and recommendations | No public health/EOH decision-framework |
| Hodge 2011 | Building evidence for legal decision making in real time: legal triage in public health emergencies. | No public health/EOH decision-framework |
| Holman 2017 | Part I--Comparing Noncancer Chronic Human Health Reference Values: An Analysis of Science Policy Choices. | Hazard/risk identification framework |
| Holman 2017 | Part II: Quantitative Evaluation of Choices Used in Setting Noncancer Chronic Human Health Reference Values Across Organizations. | No public health/EOH decision-framework |
| Hopkins 2011 | Risk-management and rule-compliance: Decision-making in hazardous industries | No public health/EOH decision-framework |
| Horne 2018 | Informing Environmental Water Management Decisions: Using Conditional Probability Networks to Address the Information Needs of Planning and Implementation Cycles. | No public health/EOH decision-framework |
| Hovell 2011 | Smokefree community policies promote home smoking bans. Unknown mechanisms and opportunities for preventive medicine. | No public health/EOH decision-framework |
| Howard 2017 | Using systematic review in occupational safety and health. | Evidence appraisal framework |
| Hoyos 2015 | Incorporating environmental attitudes in discrete choice models: An exploration of the utility of the awareness of consequences scale | No public health/EOH decision-framework |
| Hristozov 2016 | Demonstration of a modelling-based multi-criteria decision analysis procedure for prioritisation of occupational risks from manufactured nanomaterials. | No public health/EOH decision-framework |
| Huang 2012 | Cumulative environmental vulnerability and environmental justice in California's San Joaquin Valley. | Hazard/risk identification framework |
| Iavicoli 2011 | Occupational health and safety policy and psychosocial risks in Europe: the role of stakeholders' perceptions. | No public health/EOH decision-framework |
| Iavicoli 2019 | New avenues for prevention of occupational cancer: a global policy perspective. | No public health/EOH decision-framework |
| Ingram 2020 | Health Disparities, Transportation Equity and Complete Streets: a Case Study of a Policy Development Process through the Lens of Critical Race Theory | No public health/EOH decision-framework |
| J 2019 | A nuanced approach to the Environmental Noise Guidelines for the European Region related to traffic noise. | No public health/EOH decision-framework |
| Jennings 2012 | Worldwide regulatory guidance values for surface soil exposure to carcinogenic or mutagenic polycyclic aromatic hydrocarbons. | No public health/EOH decision-framework |
| Jiang 2018 | Formal and informal environmental sensing data and integration potential: Perceptions of citizens and experts. | No public health/EOH decision-framework |
| Jolly 2015 | Work-Related Asthma. | No public health/EOH decision-framework |
| Kaminsky 2015 | Cultured construction: global evidence of the impact of national values on sanitation infrastructure choice. | No public health/EOH decision-framework |
| Kassotis 2020 | Endocrine-disrupting chemicals: economic, regulatory, and policy implications. | No public health/EOH decision-framework |
| Kelsall 2015 | Don't ask, don't tell: Canadian policies on radon. | No public health/EOH decision-framework |
| Kienzler 2016 | Regulatory assessment of chemical mixtures: Requirements, current approaches and future perspectives. | No public health/EOH decision-framework |
| Kiran 2019 | The development of a globally acceptable national model for occupational hygiene in Turkey: a modified Delphi study. | No public health/EOH decision-framework |
| Koh 2017 | Combining Lead Exposure Measurements and Experts' Judgment Through a Bayesian Framework. | No public health/EOH decision-framework |
| Korfmacher 2014 | Health impact assessment of urban waterway decisions. | No public health/EOH decision-framework |
| Kreger 2011 | Creating an environmental justice framework for policy change in childhood asthma: a grassroots to treetops approach. | No public health/EOH decision-framework |
| Krimsky 2017 | The unsteady state and inertia of chemical regulation under the US Toxic Substances Control Act. | No public health/EOH decision-framework |
| Kurth 2018 | Decision making for independent municipal action. | No public health/EOH decision-framework |
| Landrigan 2011 | Children's vulnerability to toxic chemicals: a challenge and opportunity to strengthen health and environmental policy. | No public health/EOH decision-framework |
| Leech 2016 | Inequitable Chronic Lead Exposure: A Dual Legacy of Social and Environmental Injustice. | No public health/EOH decision-framework |
| Lieberman 2013 | Structural approaches to health promotion: what do we need to know about policy and environmental change?. | No public health/EOH decision-framework |
| Liebman 2013 | Occupational health policy and immigrant workers in the agriculture, forestry, and fishing sector. | No public health/EOH decision-framework |
| Linkov 2015 | From "weight of evidence" to quantitative data integration using multicriteria decision analysis and Bayesian methods. | No public health/EOH decision-framework |
| Lis 2017 | How to Choose? Using the Delphi Method to Develop Consensus Triggers and Indicators for Disaster Response. | No public health/EOH decision-framework |
| Litow 2015 | Occupational Interstitial Lung Diseases. | No public health/EOH decision-framework |
| Liu 2011 | Guided adaptive optimal decision making approach for uncertainty based watershed scale load reduction. | No public health/EOH decision-framework |
| Liu 2012 | Facilitating knowledge transfer: decision support tools in environment and health. | No public health/EOH decision-framework |
| London 2014 | Ethics in occupational health: deliberations of an international workgroup addressing challenges in an African context. | No public health/EOH decision-framework |
| Lopez-Alonso 2020 | Management of occupational risk prevention of nanomaterials manufactured in construction sites in the eu | Hazard/risk identification framework |
| Loring 2011 | Managing environmental risks: the benefits of a place-based approach. | No public health/EOH decision-framework |
| Lu 2015 | Optimization-based multicriteria decision analysis for identification of desired petroleum-contaminated groundwater remediation strategies. | No public health/EOH decision-framework |
| Mac 2019 | Examining Agricultural Workplace Micro and Macroclimate Data Using Decision Tree Analysis to Determine Heat Illness Risk. | Hazard/risk identification framework |
| MacEachen 2016 | Systematic review of qualitative literature on occupational health and safety legislation and regulatory enforcement planning and implementation. | No public health/EOH decision-framework |
| Macnaughton 2013 | Bringing politics and evidence together: policy entrepreneurship and the conception of the At Home/Chez Soi Housing First Initiative for addressing homelessness and mental illness in Canada. | No public health/EOH decision-framework |
| Mahapatra 2014 | The need for evidence-based public health response in disasters. | No public health/EOH decision-framework |
| Maier 2014 | Derivation of an occupational exposure limit for inorganic borates using a weight of evidence approach. | No public health/EOH decision-framework |
| Makov 2020 | Inconsistent allocations of harms versus benefits may exacerbate environmental inequality. | No public health/EOH decision-framework |
| Malachowski 2017 | The Sociopolitical Context of Canada's National Standard for Psychological Health and Safety in the Workplace: Navigating Policy Implementation. | No public health/EOH decision-framework |
| Marinucci 2014 | Building Resilience Against Climate Effects-a novel framework to facilitate climate readiness in public health agencies. | No public health/EOH decision-framework |
| McCarty 2018 | The regulatory challenge of chemicals in the environment: Toxicity testing, risk assessment, and decision-making models. | No public health/EOH decision-framework |
| Mederake 2019 | Shaping EU Plastic Policies: The Role of Public Health vs. Environmental Arguments. | No public health/EOH decision-framework |
| Mera 2015 | A Public health decision support system model using reasoning methods | No public health/EOH decision-framework |
| Messinger 2014 | An approach for the delineation of a generic cut-off value for local respiratory tract irritation by irritating or corrosive substances as a pragmatic tool to fulfill REACH requirements. | Hazard/risk identification framework |
| Milillo 2012 | Use of geostatistics for remediation planning to transcend urban political boundaries. | No public health/EOH decision-framework |
| Mohler 2012 | RACER: dynamic use of environmental measurement data for decision making and communication. | No public health/EOH decision-framework |
| Mohring 2019 | Quantity based indicators fail to identify extreme pesticide risks. | Hazard/risk identification framework |
| Money 2016 | Wishful Thinking? Inside the Black Box of Exposure Assessment. | No public health/EOH decision-framework |
| Moore 2017 | Application of ecosystem services in natural resource management decision making. | No public health/EOH decision-framework |
| Moretti 2017 | Environmental, Human Health and Socio-Economic Effects of Cement Powders: The Multicriteria Analysis as Decisional Methodology. | No public health/EOH decision-framework |
| Morfeld 2014 | Threshold value estimation for respirable quartz dust exposure and silicosis incidence among workers in the German porcelain industry. | No public health/EOH decision-framework |
| Morgan 2019 | A risk of bias instrument for non-randomized studies of exposures: A users' guide to its application in the context of GRADE. | Evidence appraisal framework |
| Moridi 2017 | Selection of optimized air pollutant filtration technologies for petrochemical industries through multiple-attribute decision-making. | No public health/EOH decision-framework |
| Morodi 2018 | Environmental Decision Making on Acid Mine Drainage Issues in South Africa: An Argument for the Precautionary Principle. | No public health/EOH decision-framework |
| Morrice 2013 | Coal mining, social injustice and health: a universal conflict of power and priorities. | No public health/EOH decision-framework |
| Msibi 2018 | Using e-Delphi to formulate and appraise the guidelines for women's health concerns at a coal mine: A case study. | No public health/EOH decision-framework |
| Neira 2017 | Environmental health policies for women's, children's and adolescents' health. | No public health/EOH decision-framework |
| Nelms 2019 | Evaluating potential refinements to existing Threshold of Toxicological Concern (TTC) values for environmentally-relevant compounds. | No public health/EOH decision-framework |
| Nielsen 2017 | Evaluation of airborne sensory irritants for setting exposure limits or guidelines: A systematic approach. | Hazard/risk identification framework |
| Nweke 2011 | Symposium on integrating the science of environmental justice into decision-making at the Environmental Protection Agency: an overview. | No public health/EOH decision-framework |
| Nweke 2011 | A framework for integrating environmental justice in regulatory analysis. | No public health/EOH decision-framework |
| Ozdemir 2020 | Decision-making for the selection of different leachate treatment/management methods: the ANP and PROMETHEE approaches. | No public health/EOH decision-framework |
| Palmer Fry 2017 | Monitoring local well-being in environmental interventions: A consideration of practical trade-offs | No public health/EOH decision-framework |
| Pennell 2013 | Bridging research and environmental regulatory processes: the role of knowledge brokers. | No public health/EOH decision-framework |
| Pieper 2019 | Understanding lead in water and avoidance strategies: a United States perspective for informed decision-making. | No public health/EOH decision-framework |
| Pirkle 2016 | Managing mercury exposure in northern Canadian communities. | No public health/EOH decision-framework |
| Pizzol 2011 | Regional risk assessment for contaminated sites part 2: ranking of potentially contaminated sites. | No public health/EOH decision-framework |
| Pizzol 2015 | Risk-based prioritization methodology for the classification of groundwater pollution sources | No public health/EOH decision-framework |
| Post 2017 | Key scientific issues in developing drinking water guidelines for perfluoroalkyl acids: Contaminants of emerging concern. | No public health/EOH decision-framework |
| Pottage 2014 | Responding to biological incidents - What are the current issues in remediation of the contaminated environment? | No public health/EOH decision-framework |
| Pruvot 2019 | Toward a quantification of risks at the nexus of conservation and health: The case of bushmeat markets in Lao PDR. | Hazard/risk identification framework |
| Rahimdel 2020 | Prioritization of practical solutions for the vibrational health risk reduction of mining trucks using fuzzy decision making. | No public health/EOH decision-framework |
| Reinikainen 2016 | Promoting justified risk-based decisions in contaminated land management. | No public health/EOH decision-framework |
| Reis 2019 | Decision-making under uncertainty in environmental health policy: new approaches. | No public health/EOH decision-framework |
| Ronen 2012 | Rationales behind irrationality of decision making in groundwater quality management. | No public health/EOH decision-framework |
| Rooney 2014 | Systematic review and evidence integration for literature-based environmental health science assessments. | Evidence appraisal framework |
| Saeed 2012 | Environmental impact assessment (EIA): an overlooked instrument for sustainable development in Pakistan. | Hazard/risk identification framework |
| Saldavar-Tanaka 2021 | Should the precautionary principle be implemented in Europe with regard to nanomaterials? Expert interviews | No public health/EOH decision-framework |
| Samantra 2017 | A risk-based decision support framework for selection of appropriate safety measure system for underground coal mines. | No public health/EOH decision-framework |
| Samuels 2017 | Pathways to Housing Policy: Translating Research to Policy to Achieve Impact on Well Being. | No public health/EOH decision-framework |
| Scheepers 2011 | Application of biological monitoring for exposure assessment following chemical incidents: a procedure for decision making. | No public health/EOH decision-framework |
| Schenk 2019 | Industry Derived Occupational Exposure Limits: A Survey of Professionals on the Dutch System of Exposure Guidelines. | Hazard/risk identification framework |
| Schoch-Spana 2020 | Vector control in Zika-affected communities: Local views on community engagement and public health ethics during outbreaks | No public health/EOH decision-framework |
| Scholten 2014 | Strategic rehabilitation planning of piped water networks using multi-criteria decision analysis. | No public health/EOH decision-framework |
| Shaffer 2017 | Developing the Regulatory Utility of the Exposome: Mapping Exposures for Risk Assessment through Lifestage Exposome Snapshots (LEnS). | No public health/EOH decision-framework |
| Sharma 2014 | The legal framework to manage chemical pollution in India and the lesson from the Persistent Organic Pollutants (POPs). | No public health/EOH decision-framework |
| Shen 2016 | Vulnerability assessment of urban ecosystems driven by water resources, human health and atmospheric environment | No public health/EOH decision-framework |
| Simon 2011 | Just who is at risk? The ethics of environmental regulation. | No public health/EOH decision-framework |
| Sizirici 2011 | Knowledge based ranking algorithm for comparative assessment of post-closure care needs of closed landfills. | No public health/EOH decision-framework |
| Soderberg 2016 | Complex governance structures and incoherent policies: Implementing the EU water framework directive in Sweden. | No public health/EOH decision-framework |
| Sparrevik 2012 | Use of stochastic multi-criteria decision analysis to support sustainable management of contaminated sediments. | Evidence appraisal framework |
| Stahl 2018 | Applying theories to better understand socio-political challenges in implementing evidence-based work disability prevention strategies. | No public health/EOH decision-framework |
| Stokstad 2020 | EPA expands controversial 'transparency' plan. | Full text unavailable |
| Stolk 2017 | Decision intelligence in public health ‚Äì Dione | No public health/EOH decision-framework |
| Sutton 2013 | Risk communication and decision tools for children's health protection. | No public health/EOH decision-framework |
| Sweeney 2017 | The Role of Healthcare Professionals in Environmental Health and Fertility Decision-Making. | No public health/EOH decision-framework |
| Syberg 2016 | Environmental risk assessment of chemicals and nanomaterials--The best foundation for regulatory decision-making?. | Hazard/risk identification framework |
| Tam 2020 | Preparing for uncertainty during public health emergencies: What Canadian health leaders can do now to optimize future emergency response. | No public health/EOH decision-framework |
| Tamers 2020 | Envisioning the future of work to safeguard the safety, health, and well-being of the workforce: A perspective from the CDC's National Institute for Occupational Safety and Health. | No public health/EOH decision-framework |
| Tan 2017 | Selecting Cooking Methods to Decrease Persistent Organic Pollutant Concentrations in Food of Animal Origin Using a Consensus Decision-Making Model. | No public health/EOH decision-framework |
| Taxell 2013 | Methodology for national risk analysis and prioritization of toxic industrial chemicals. | Hazard/risk identification framework |
| Teeguarden 2016 | Completing the Link between Exposure Science and Toxicology for Improved Environmental Health Decision Making: The Aggregate Exposure Pathway Framework. | Hazard/risk identification framework |
| Terracini 2019 | Contextualising the policy decision to ban asbestos | No public health/EOH decision-framework |
| Teysseire 2019 | Identification and Prioritization of Environmental Reproductive Hazards: A First Step in Establishing Environmental Perinatal Care. | No public health/EOH decision-framework |
| Thomas 2020 | Ethical Pandemic Control Through the Public Health Code of Ethics. | No public health/EOH decision-framework |
| Trasande 2015 | Estimating burden and disease costs of exposure to endocrine-disrupting chemicals in the European union. | Economic/cost-benefit framework |
| Trasande 2016 | Burden of disease and costs of exposure to endocrine disrupting chemicals in the European Union: an updated analysis. | No public health/EOH decision-framework |
| UmstattdMeyer 2020 | Physical activity Space Methodology for Assessment and Prioritization (PASMAP): Combining systematic observations with community perceptions to identify community physical activity resource priorities | No public health/EOH decision-framework |
| vanBroekhuizen 2011 | Dealing with uncertainties in the nanotech workplace practice: making the precautionary approach operational. | No public health/EOH decision-framework |
| Vandenberg 2016 | A proposed framework for the systematic review and integrated assessment (SYRINA) of endocrine disrupting chemicals. | No public health/EOH decision-framework |
| Wang 2013 | Necessity and approach to integrated nanomaterial legislation and governance. | No public health/EOH decision-framework |
| Wang 2018 | What are the new challenges, goals, and tasks of occupational health in China's Thirteenth Five-Year Plan (13th FYP) period?. | No public health/EOH decision-framework |
| Watterson 2020 | Lagging and Flagging: Air Pollution, Shale Gas Exploration and the Interaction of Policy, Science, Ethics and Environmental Justice in England. | No public health/EOH decision-framework |
| Weinhold 2012 | EPA proposes tighter particulate air pollution standards. | Hazard/risk identification framework |
| Wolffe 2020 | A Survey of Systematic Evidence Mapping Practice and the Case for Knowledge Graphs in Environmental Health and Toxicology. | No public health/EOH decision-framework |
| Woodruff 2011 | The need for better public health decisions on chemicals released into our environment. | Hazard/risk identification framework |
| WorldHealthOrganization 2011 | Evaluation of certain contaminants in food. | No public health/EOH decision-framework |
| Xiao 2018 | A novel multi-criteria decision making method for assessing health-care waste treatment technologies based on D numbers | No public health/EOH decision-framework |
| Xie 2017 | Evaluating the Impact of the U.S. National Toxicology Program: A Case Study on Hexavalent Chromium. | No public health/EOH decision-framework |
| Yabar 2012 | Comparative assessment of the co-evolution of environmental indicator systems in Japan and China | No public health/EOH decision-framework |
| Yamauchi 2017 | Overwork-related disorders in Japan: recent trends and development of a national policy to promote preventive measures. | No public health/EOH decision-framework |
| Yasui 2015 | 250 mSv: temporary increase in the emergency exposure dose limit in response to the TEPCO Fukushima Daiichi NPP accident and its decision making process. | No public health/EOH decision-framework |
| Yu 2018 | Investigation of a Brownfield Conflict Considering the Strength of Preferences. | No public health/EOH decision-framework |
| Yuen 2013 | Using health impact assessment to integrate environmental justice into federal environmental regulatory analysis. | No public health/EOH decision-framework |
| Zabeo 2011 | Regional risk assessment for contaminated sites part 1: vulnerability assessment by multicriteria decision analysis. | Hazard/risk identification framework |
| Zartarian 2017 | Children's Lead Exposure: A Multimedia Modeling Analysis to Guide Public Health Decision-Making. | Hazard/risk identification framework |
| Zhao 2018 | A Novel Environmental Justice Indicator for Managing Local Air Pollution. | No public health/EOH decision-framework |
| Zimmer 2012 | Policy relevant results from an expert elicitation on the health risks of phthalates. | No public health/EOH decision-framework |
| Zolfagharipoor 2016 | A decision-making framework for river water quality management under uncertainty: Application of social choice rules. | No public health/EOH decision-framework |
| Zolfagharipoor 2017 | Effluent trading in river systems through stochastic decision-making process: a case study. | No public health/EOH decision-framework |

# Supplement E. GRADE Evidence to Decision (EtD) framework for health system and public health decisions

| **Criteria** | **Main questions** | **Detailed judgements** |
| --- | --- | --- |
| **Problem** | Is the problem a priority? | - Are the consequences of the problem serious (i.e. severe or important in terms of the potential benefits or savings)? - Is the problem urgent? [not relevant for coverage decisions] - Is it a recognized priority (e.g. based on a political or policy decision)? [Not relevant when an individual patient perspective is taken] |
| **Desirable effects** | How substantial are the desirable anticipated effects? | Judgments for each outcome for which there is a desirable effect |
| **Undesirable effects** | How substantial are the undesirable anticipated effects? | Judgments for each outcome for which there is an undesirable effect |
| **Certainty of evidence** | What is the overall certainty of the evidence of effects? | See GRADE guidance regarding detailed judgments about the quality of evidence or certainty in estimates of effects |
| **Values** | Is there important uncertainty about or variability in how much people value the main outcomes? | - Is there important uncertainty about how much people value each of the main outcomes? - Is there important variability in how much people value each of the main outcomes? [not relevant for coverage decisions] |
| **Balance of effects** | Does the balance between desirable and undesirable effects favor the intervention or the comparison? | - Judgments regarding each of the four preceding criteria - To what extent do the following considerations influence the balance between the desirable and undesirable effects:   - How much less people value outcomes that are in the future compared to outcomes that occur now (their discount rates)   - People’s attitudes towards undesirable effects (how risk averse they are)   - People’s attitudes towards desirable effects (how risk seeking they are) |
| **Resources required** | How large are the resource requirements (costs)? | - How large is the difference in each item of resource use for which fewer resources are required? - How large is the difference in each item of resource use for which more resources are required? |
| **Certainty of evidence of required resources** | What is the certainty of the evidence of resource requirements? | - Have all-important items of resource use that may differ between the options being considered been identified? - How certain is the evidence of differences in resource use between the options being considered? (see GRADE guidance regarding detailed judgments about the quality of evidence or certainty in estimates) - How certain is the cost of the items of resource use that differ between the options being considered? - Is there important variability in the cost of the items of resource use that differ between the options being considered? |
| **Cost effectiveness** | Does the cost effectiveness of the intervention favor the intervention or the comparison? | - Judgments regarding each of the six preceding criteria - Is the cost-effectiveness ratio sensitive to one-way sensitivity analyses? - Is the cost-effectiveness ratio sensitive to multi-variable sensitivity analyses? - Is the economic evaluation on which the cost-effectiveness estimate is based reliable? - Is the economic evaluation on which the cost-effectiveness estimate is based applicable to the setting(s) of interest? |
| **Equity** | What would be the impact on health equity? | - Are there groups or settings that might be disadvantaged in relation to the problem or options that are considered? - Are there plausible reasons for anticipating differences in the relative effectiveness of the option for disadvantaged groups or settings? - Are there different baseline conditions across groups or settings that affect the absolute effectiveness of the intervention or the importance of the problem for disadvantaged groups or settings? - Are there important considerations that should be made when implementing the intervention in order to ensure that inequities are reduced, if possible, and that they are not increased? |
| **Acceptability** | Is the intervention acceptable to key stakeholders? | - Are there key stakeholders that would not accept the distribution of the benefits, harms and costs? - Are there key stakeholders that would not accept the costs or undesirable effects in the short term for desirable effects (benefits) in the future? - Are there key stakeholders that would not agree with the values attached to the desirable or undesirable effects (because of how they might be affected personally or because of their perceptions of the relative importance of the effects for others)? - Would the intervention adversely affect people’s autonomy? - Are there key stakeholders that would disapprove of the intervention morally, for reasons other than its effects on people’s autonomy (e.g. in relation to ethical principles such as no maleficence, beneficence or justice)? |
| **Feasibility** | Is the intervention feasible to implement? | **For decisions other than coverage decisions:**   - Is the intervention or option sustainable? - Are there important barriers that are likely to limit the feasibility of implementing the intervention (option) or require consideration when implementing it?   **For coverage decisions:**   - Is coverage of the intervention sustainable? - Is it feasible to ensure appropriate use for approved indications? - Is inappropriate use (indications that are not approved) an important concern? - Is there capacity to meet increased demand if covered? - Are there important legal or bureaucratic or ethical constraints that make it difficult or impossible to cover the intervention? |

# Supplement E. Unique EOH decision factors organized by related GRADE EtD assessment criteria

| **Assessment Criteria** | **Discovered EOH Decision Considerations** |
| --- | --- |
| **Problem** | - The decision might lead to irreversible and severe consequences and the values at stake are also irreplaceable - Is the problem urgent? - Does the problem constitute an extraordinary event? - Is it a recognized priority (e.g., based on a political or policy decision)? - Local priorities: extent to which funding for intervention is a city or county priority compared with other rivalling priorities - Decisions by other jurisdictions: what have other jurisdictions (provinces, countries) done with respect to the technology, device, or intervention being considered? - Requires a coordinated international response - Magnitude of need (availability of an effective, comparable alternative) - Origin of/reason for request: Who requested the review? What was their rationale? What is their responsibility/mandate? - Availability of data: extent to which potential health risk factors are monitored - Availability of data: hazard inventory and information on severity of hazards - Availability of data: whether contamination of water and soil is known to be present in the community - Community involvement: extent to which the unserved community has organized and advocated for an intervention - Decisions can be enforced by means of laws or regulations - How regulated is the intervention: are there penalties for failure? - Unique toxicological considerations (i.e., developmental, epigenetic) - Unique exposure considerations (i.e., persistence, bioaccumulation) - Chemical properties: ADME (Absorption, Distribution, Metabolism, and Elimination) |
| **Desirable effects** | - Time span for the alternative policies and measures to reach their full effectiveness - It is more important to avoid false negatives than false positives - Does early intervention of this exposure improve outcomes compared to later intervention? - Timing is at least as important as being right - Most proportional response, if compulsion or coercion is needed |
| **Undesirable effects** | - It is more important to avoid false negatives than false positives - Timing is at least as important as being right |
| **Certainty of evidence** | None |
| **Values** | - Stakeholders: types and distribution of types, identification of new groups of stakeholders - Values that tend to be systematically downplayed by traditional decision methods - Social justice and equality: How is social justice and/or equality addressed? What is the duration of the intervention and are there issues of intergenerational equity? Are the impacts/benefits unreasonably disproportionate to particular groups? Is spirit of ‘polluter pays principle’ upheld with regard to distribution of impacts/benefits? - Expected societal values: broadly shared values in society that bear on the appropriate use of the intervention - Interactions with different branches and levels of government, as well as the citizens that they represent - Community engagement |
| **Balance of effects** | - People’s attitudes towards desirable effects (how risk seeking they are). - People’s attitudes towards undesirable effects (how risk averse they are). |
| **Resources required** | None |
| **Certainty of evidence of required resources** | None |
| **Cost effectiveness** | - Cost-effectiveness with a societal perspective and using average costs - Differential cost analysis process, budget impact analysis, cost-effectiveness using hospital perspective (i.e. actual costs for hospital) - Economic evaluation: a measure of the net cost or efficiency of the intervention compared to other alternatives; the uncertainty of results should be considered - Costs, benefits and impacts of potential actions - Adequacy: appropriateness of cost and outcome measures, comprehensiveness of cost and outcome valuation/aggregation |
| **Equity** | - The minimal infringement of moral considerations should have priority among other effective policies. - Expected ethical values: the potential ethical issues inherent in using or not using the technology; relevant ethical issues should be listed - Accordance with universal human rights standards. - The probable public health benefits should outweigh the infringed moral considerations. - Examining the health outcomes and legal and ethical considerations |
| **Acceptability** | - Would the intervention (option) adversely affect people’s autonomy? - Are there key stakeholders that would disapprove of the intervention (option) morally, for reasons other than its effects on people’s autonomy (i.e., in relationship to ethical principles such as non-maleficence, beneficence or justice)? - Most proportional response, if compulsion or coercion is needed - Accordance with universal human rights standards. - Protect human rights and individual autonomy - Congressional mandate: are congressional views or mandates going to change? - Awareness of health risks: extent to which decision-makers are aware of health risks associated with the exposure - Communication: quality of communication plan - Decisions can be enforced by means of laws or regulations - Examining the health outcomes and legal and ethical considerations |
| **Feasibility** | - Federal laws and regulations now enable alternative uses for the exposure or intervention - Regulatory framework: Current laws and regulations applying to worker and public health and safety - Congressional mandate: are congressional views or mandates going to change? |

# Supplement F. Round 1 Delphi rating instrument

| **GRADE Evidence-to-Decision (EtD) Framework for Environmental and Occupational Health** | | | | | | | | | | |
| --- | --- | --- | --- | --- | --- | --- | --- | --- | --- | --- |
| **General instructions** | | | | | | | | | | |
| **Please indicate your agreement on scale of 1 to 7.** In column C, **1** indicates that you **strongly disagree** that the criterion should be included. **7** indicates that you **strongly agree** that the criterion should be included.  In column D, **1** indicates that your **strongly disagree** with the wording, and **7** that you **strongly agree** with the wording.  In column E, please indicate any suggested revisions.  In column F, please add any other comments or thoughts about the criterion or guidance.  **At this stage, the number of questions in the questionnaire should be irrelevant to your answers.** | | | | | | | | | | |
| **Criteria and guidance** | | | | | | | | | | |
| **Scoping criteria** | **Guidance for the scoping criteria** | | **Should this scoping criterion be included in scoping an EOH recommendation?** | | **Is the guidance for the scoping criterion worded appropriately for scoping an EOH recommendation?** | | **What revisions, if any, would you recommend for the wording of the criterion and/or guidance?** | | **Do you have any other thoughts or comments on the criteria or guidance?** | |
| **Purpose** | Specify health intents (i.e., prevention, diagnosis, treatment, etc.) and expected benefits or outcomes. E.g. preventing thromboembolic complications of patients undergoing elective orthopedic surgery. | | 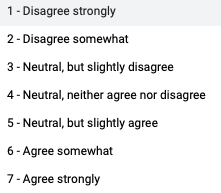 | | 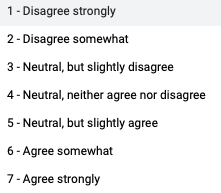 | | [[FREE TEXT RESPONSE]] | | [[FREE TEXT RESPONSE]] | |
| **Perspective** | Specify the perspective that the panel will take when making recommendations: this of an individual patients, their families, providers caring for those patients, public health, health system, payer, population (the society), etc. | | [[see above]] | | [[see above]] | | [[see above]] | | [[see above]] | |
| **Target population** | Specify subjects to whom those recommendations apply (i.e. patients, society, etc.) E.g. adults undergoing elective orthopedic surgery, all women 40 years of age or older, etc. | | [[see above]] | | [[see above]] | | [[see above]] | | [[see above]] | |
| **Setting** | Specify level of health care (i.e. primary, high, etc.) where these recommendations are supposed to be implemented. | | [[see above]] | | [[see above]] | | [[see above]] | | [[see above]] | |
| **Key coexisting conditions** | Specify the key coexisting conditions (comorbidities) that might need to be considered when making recommendations. E.g. patients with COPD frequently have coexisting heart failure and diabetes that may influence the choice of the optimal management. | | [[see above]] | | [[see above]] | | [[see above]] | | [[see above]] | |
| **Types of interventions** | Specify which preventive, therapeutic and diagnostic interventions will be covered and which will be not. | | [[see above]] | | [[see above]] | | [[see above]] | | [[see above]] | |
| **Key stakeholders / users** | Specify all relevant professional groups, institutions, patients, public, etc. who are target users or beneficiaries of these guidelines and/or whose views should be sought | | [[see above]] | | [[see above]] | | [[see above]] | | [[see above]] | |
| **Key resources** | Specify resources needed for the implementation of guidelines (i.e. need for additional human resources, equipment, infrastructure, system changes, etc.) and potential barriers to implementation. | | [[see above]] | | [[see above]] | | [[see above]] | | [[see above]] | |
| **Key implementation issues** | --- | | [[see above]] | | [[see above]] | | [[see above]] | | [[see above]] | |
| **Existing documents** | List all existing documents/guidelines on the same or similar topic that are likely to be currently used in practice (e.g. guidelines developed by other organizations). | | [[see above]] | | [[see above]] | | [[see above]] | | [[see above]] | |
| **Suggestions for additional guidance** | | | | | | | | | | |
| Is there any additional guidance or themes that you think would be relevant to scoping environmental health policy recommendations, that are missing from the above? Please indicate these to the right. | | | [[FREE TEXT RESPONSE]] | | | | | | | |
| **Any other comments** | | | | | | | | | | |
| Please add any additional thoughts or comments not covered by any of the above. | | | [[FREE TEXT RESPONSE]] | |  | |  | |  | |
| **Criteria and detailed judgements** | | | | | | | | | | |
| **Decision framework criteria** | | **Items (detailed judgements)** | | **Should this detailed judgement be included in developing an EOH recommendation?** | | **Is the wording for the detailed judgement appropriate for developing an EOH recommendation?** | | **What revisions, if any, would you recommend for the wording of the detailed judgement?** | | **Do you have any other thoughts or comments on the detailed judgement?** |
| **Problem**  ***Is the problem a priority?*** | | Are the consequences of the problem serious (i.e. severe or important in terms of the potential benefits or savings)? | | 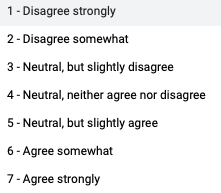 | | 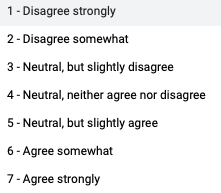 | | [[FREE TEXT RESPONSE]] | | [[FREE TEXT RESPONSE]] |
|  |  | The decision might lead to irreversible and severe consequences and the values at stake are also irreplaceable | | [[see above]] | | [[see above]] | | [[see above]] | | [[see above]] |
|  |  | Is the problem urgent? | | [[see above]] | | [[see above]] | | [[see above]] | | [[see above]] |
|  |  | Does the problem constitute an extraordinary event? | | [[see above]] | | [[see above]] | | [[see above]] | | [[see above]] |
|  |  | Is it a recognized priority (e.g. based on a political or policy decision)? | | [[see above]] | | [[see above]] | | [[see above]] | | [[see above]] |
|  |  | Local priorities: extent to which funding for intervention is a city or county priority compared with other rivaling priorities | | [[see above]] | | [[see above]] | | [[see above]] | | [[see above]] |
|  |  | Decisions by other jurisdictions: what have other jurisdictions (provinces, countries) done with respect to the technology, device, or intervention being considered? | | [[see above]] | | [[see above]] | | [[see above]] | | [[see above]] |
|  |  | Requires a coordinated international response | | [[see above]] | | [[see above]] | | [[see above]] | | [[see above]] |
|  |  | Magnitude of need (availability of an effective, comparable alternative) | | [[see above]] | | [[see above]] | | [[see above]] | | [[see above]] |
|  |  | Origin of/reason for request: Who requested the review? What was their rationale? What is their responsibility/mandate? | | [[see above]] | | [[see above]] | | [[see above]] | | [[see above]] |
|  |  | Availability of data: extent to which potential health risk factors are monitored | | [[see above]] | | [[see above]] | | [[see above]] | | [[see above]] |
|  |  | Availability of data: hazard inventory and information on severity of hazards | | [[see above]] | | [[see above]] | | [[see above]] | | [[see above]] |
|  |  | Availability of data: whether contamination of water and soil is known to be present in the community | | [[see above]] | | [[see above]] | | [[see above]] | | [[see above]] |
|  |  | Community involvement: extent to which the unserved community has organized and advocated for an intervention | | [[see above]] | | [[see above]] | | [[see above]] | | [[see above]] |
|  |  | Decisions can be enforced by means of laws or regulations | | [[see above]] | | [[see above]] | | [[see above]] | | [[see above]] |
|  |  | How regulated is the intervention: are there penalties for failure? | | [[see above]] | | [[see above]] | | [[see above]] | | [[see above]] |
|  |  | Unique toxicological considerations (i.e., developmental, epigenetic) | | [[see above]] | | [[see above]] | | [[see above]] | | [[see above]] |
|  |  | Unique exposure considerations (i.e., persistence, bioaccumulation) | | [[see above]] | | [[see above]] | | [[see above]] | | [[see above]] |
|  |  | Chemical properties: ADME (Absorption, Distribution, Metabolism, and Elimination) | | [[see above]] | | [[see above]] | | [[see above]] | | [[see above]] |
| **Desirable effects**  ***How substantial are the desirable anticipated effects?*** | | How substantial is the anticipated effect (difference) for each main outcome for which there is a desirable effect? | | [[see above]] | | [[see above]] | | [[see above]] | | [[see above]] |
|  |  | Time span for the alternative policies and measures to reach their full effectiveness | | [[see above]] | | [[see above]] | | [[see above]] | | [[see above]] |
|  |  | It is more important to avoid false negatives than false positives | | [[see above]] | | [[see above]] | | [[see above]] | | [[see above]] |
|  |  | Does early intervention of this exposure improve outcomes compared to later intervention? | | [[see above]] | | [[see above]] | | [[see above]] | | [[see above]] |
|  |  | Timing is at least as important as being right | | [[see above]] | | [[see above]] | | [[see above]] | | [[see above]] |
|  |  | Most proportional response, if compulsion or coercion is needed | | [[see above]] | | [[see above]] | | [[see above]] | | [[see above]] |
| **Undesirable effects**  ***How substantial are the undesirable anticipated effects?*** | | How substantial is the anticipated effect (difference) for each main outcome for which there is an undesirable effect? | | [[see above]] | | [[see above]] | | [[see above]] | | [[see above]] |
|  |  | It is more important to avoid false negatives than false positives | | [[see above]] | | [[see above]] | | [[see above]] | | [[see above]] |
|  |  | Timing is at least as important as being right | | [[see above]] | | [[see above]] | | [[see above]] | | [[see above]] |
| **Certainty of evidence**  ***What is the overall certainty of the evidence of effects?*** | | - | | [[see above]] | | [[see above]] | | [[see above]] | | [[see above]] |
| **Values**  ***Is there important uncertainty about or variability in how much people value the main outcomes?*** | | Is there important uncertainty about how much people value the main outcomes? | | [[see above]] | | [[see above]] | | [[see above]] | | [[see above]] |
|  |  | Stakeholders: types and distribution of types, identification of new groups of stakeholders | | [[see above]] | | [[see above]] | | [[see above]] | | [[see above]] |
|  |  | Values that tend to be systematically downplayed by traditional decision methods | | [[see above]] | | [[see above]] | | [[see above]] | | [[see above]] |
|  |  | Social justice and equality: How is social justice and/or equality addressed? What is the duration of the intervention and are there issues of intergenerational equity? Are the impacts/benefits unreasonably disproportionate to particular groups? Is spirit of ‘polluter pays principle’ upheld with regard to distribution of impacts/benefits? | | [[see above]] | | [[see above]] | | [[see above]] | | [[see above]] |
|  |  | Expected societal values: broadly shared values in society that bear on the appropriate use of the intervention | | [[see above]] | | [[see above]] | | [[see above]] | | [[see above]] |
|  |  | Interactions with different branches and levels of government, as well as the citizens that they represent | | [[see above]] | | [[see above]] | | [[see above]] | | [[see above]] |
|  |  | Community engagement | | [[see above]] | | [[see above]] | | [[see above]] | | [[see above]] |
| **Balance of effects**  ***Does the balance between desirable and undesirable effects favor the intervention or the comparison?*** | | Is there important uncertainty about or variability in how much people value the main outcomes? | | [[see above]] | | [[see above]] | | [[see above]] | | [[see above]] |
|  |  | How much less people value outcomes that are in the future compared to outcomes that occur now (their discount rates) | | [[see above]] | | [[see above]] | | [[see above]] | | [[see above]] |
|  |  | What is the overall certainty of the evidence of effects? | | [[see above]] | | [[see above]] | | [[see above]] | | [[see above]] |
|  |  | How substantial are the desirable anticipated effects? | | [[see above]] | | [[see above]] | | [[see above]] | | [[see above]] |
|  |  | People’s attitudes towards desirable effects (how risk seeking they are). | | [[see above]] | | [[see above]] | | [[see above]] | | [[see above]] |
|  |  | How substantial are the undesirable anticipated effects? | | [[see above]] | | [[see above]] | | [[see above]] | | [[see above]] |
|  |  | People’s attitudes towards undesirable effects (how risk averse they are). | | [[see above]] | | [[see above]] | | [[see above]] | | [[see above]] |
| **Resources required**  ***How large are the resource requirements (costs)?*** | | How large is the difference in each item of resource for which fewer resources are required? | | [[see above]] | | [[see above]] | | [[see above]] | | [[see above]] |
|  |  | How large is the difference in each item of resource use for which more resources are required? | | [[see above]] | | [[see above]] | | [[see above]] | | [[see above]] |
| **Certainty of evidence of resources required**  ***What is the certainty of the evidence of resource requirements (costs)?*** | | Have all important items of resource use that may differ between the options being considered been identified? | | [[see above]] | | [[see above]] | | [[see above]] | | [[see above]] |
|  |  | How certain is the cost of the items of resource use that differ between the options being considered? | | [[see above]] | | [[see above]] | | [[see above]] | | [[see above]] |
|  |  | Is there important variability in the cost of the items of resource use that differ between the options being considered? | | [[see above]] | | [[see above]] | | [[see above]] | | [[see above]] |
| **Cost effectiveness**  **Does the cost-effectiveness of the intervention favor the intervention or the comparison?** | | Is the cost-effectiveness ratio sensitive to one-way sensitivity analyses? | | [[see above]] | | [[see above]] | | [[see above]] | | [[see above]] |
|  |  | Is the cost-effectiveness ratio sensitive to multi-variable sensitivity analysis? | | [[see above]] | | [[see above]] | | [[see above]] | | [[see above]] |
|  |  | Is the economic evaluation on which the cost-effectiveness estimate is based reliable? | | [[see above]] | | [[see above]] | | [[see above]] | | [[see above]] |
|  |  | Is the economic evaluation on which the cost-effectiveness estimate is based applicable to the setting(s) of interest? | | [[see above]] | | [[see above]] | | [[see above]] | | [[see above]] |
|  |  | Cost-effectiveness with a societal perspective and using average costs | | [[see above]] | | [[see above]] | | [[see above]] | | [[see above]] |
|  |  | Differential cost analysis process, budget impact analysis, cost-effectiveness using hospital perspective (i.e. actual costs for hospital) | | [[see above]] | | [[see above]] | | [[see above]] | | [[see above]] |
|  |  | Economic evaluation: a measure of the net cost or efficiency of the intervention compared to other alternatives; the uncertainty of results should be considered | | [[see above]] | | [[see above]] | | [[see above]] | | [[see above]] |
|  |  | Costs, benefits and impacts of potential actions | | [[see above]] | | [[see above]] | | [[see above]] | | [[see above]] |
|  |  | Adequacy: appropriateness of cost and outcome measures, comprehensiveness of cost and outcome valuation/aggregation | | [[see above]] | | [[see above]] | | [[see above]] | | [[see above]] |
| **Equity**  ***What would be the impact on health equity?*** | | Are there groups or settings that might be disadvantaged in relation to the problem or options that are considered? | | [[see above]] | | [[see above]] | | [[see above]] | | [[see above]] |
|  |  | Are there plausible reasons for anticipating differences in the relative effectiveness of the option for disadvantaged groups or settings? | | [[see above]] | | [[see above]] | | [[see above]] | | [[see above]] |
|  |  | Are there different baseline conditions across groups or settings that affect the absolute effectiveness of the option or the importance of the problem for disadvantaged groups or settings? | | [[see above]] | | [[see above]] | | [[see above]] | | [[see above]] |
|  |  | Are there important considerations that should be made when implementing the intervention (option) in order to ensure that inequities are reduced, if possible, and that they are not increased? | | [[see above]] | | [[see above]] | | [[see above]] | | [[see above]] |
|  |  | The minimal infringement of moral considerations should have priority among other effective policies. | | [[see above]] | | [[see above]] | | [[see above]] | | [[see above]] |
|  |  | Expected ethical values: the potential ethical issues inherent in using or not using the technology; relevant ethical issues should be listed | | [[see above]] | | [[see above]] | | [[see above]] | | [[see above]] |
|  |  | Accordance with universal human rights standards. | | [[see above]] | | [[see above]] | | [[see above]] | | [[see above]] |
|  |  | The probable public health benefits should outweigh the infringed moral considerations. | | [[see above]] | | [[see above]] | | [[see above]] | | [[see above]] |
|  |  | Examining the health outcomes and legal and ethical considerations | | [[see above]] | | [[see above]] | | [[see above]] | | [[see above]] |
| **Acceptability**  ***Is the intervention acceptable to key stakeholders?*** | | Are there key stakeholders that would not accept the distribution of the benefits, harms and costs? | | [[see above]] | | [[see above]] | | [[see above]] | | [[see above]] |
|  |  | Are there key stakeholders that would not accept the costs or undesirable effects in the short term for desirable effects (benefits) in the future? | | [[see above]] | | [[see above]] | | [[see above]] | | [[see above]] |
|  |  | Are there key stakeholders that would not agree with the values attached to the desirable or undesirable effects (because of how they might be affected personally or because of their perceptions of the relative importance of the effects for others)? | | [[see above]] | | [[see above]] | | [[see above]] | | [[see above]] |
|  |  | Would the intervention (option) adversely affect people’s autonomy? | | [[see above]] | | [[see above]] | | [[see above]] | | [[see above]] |
|  |  | Are there key stakeholders that would disapprove of the intervention (option) morally, for reasons other than its effects on people’s autonomy (i.e. in relationship to ethical principles such as non-maleficence, beneficence or justice)? | | [[see above]] | | [[see above]] | | [[see above]] | | [[see above]] |
|  |  | Most proportional response, if compulsion or coercion is needed | | [[see above]] | | [[see above]] | | [[see above]] | | [[see above]] |
|  |  | Accordance with universal human rights standards. | | [[see above]] | | [[see above]] | | [[see above]] | | [[see above]] |
|  |  | Protect human rights and individual autonomy | | [[see above]] | | [[see above]] | | [[see above]] | | [[see above]] |
|  |  | Congressional mandate: are congressional views or mandates going to change? | | [[see above]] | | [[see above]] | | [[see above]] | | [[see above]] |
|  |  | Awareness of health risks: extent to which decision-makers are aware of health risks associated with the exposure | | [[see above]] | | [[see above]] | | [[see above]] | | [[see above]] |
|  |  | Communication: quality of communication plan | | [[see above]] | | [[see above]] | | [[see above]] | | [[see above]] |
|  |  | Decisions can be enforced by means of laws or regulations | | [[see above]] | | [[see above]] | | [[see above]] | | [[see above]] |
|  |  | Examining the health outcomes and legal and ethical considerations | | [[see above]] | | [[see above]] | | [[see above]] | | [[see above]] |
| **Feasibility**  ***Is the intervention feasible to implement?*** | | Is the intervention (option) sustainable? | | [[see above]] | | [[see above]] | | [[see above]] | | [[see above]] |
|  |  | Are there important barriers that are likely to limit the feasibility of implementing the intervention (option) or require consideration when implementing it? | | [[see above]] | | [[see above]] | | [[see above]] | | [[see above]] |
|  |  | Federal laws and regulations now enable alternative uses for the exposure or intervention | | [[see above]] | | [[see above]] | | [[see above]] | | [[see above]] |
|  |  | Regulatory framework: Current laws and regulations applying to worker and public health and safety | | [[see above]] | | [[see above]] | | [[see above]] | | [[see above]] |
|  |  | Congressional mandate: are congressional views or mandates going to change? | | [[see above]] | | [[see above]] | | [[see above]] | | [[see above]] |
| **Suggestions for additional items** | | | | | | | | | | |
| Are there any additional judgement items that you think should be considered when developing a OEH recommendation? Please indicate these to the right. | | | | [[FREE TEXT RESPONSE]] | | | | | | |
| **Any other comments** | | | | | | | | | | |
| Please add any additional thoughts or comments not covered by any of the above. | | | | [[FREE TEXT RESPONSE]] | |  | |  | |  |

# Supplement G. Round 2 Delphi rating instrument

| **GRADE Evidence-to-Decision (EtD) Framework for Environmental and Occupational Health** | | | |
| --- | --- | --- | --- |
| **General instructions - Round 2** | | | |
| **Please indicate your agreement on scale of 1 to 7.** In column C, **1** indicates that your **strongly disagree** with the wording, and **7** that you **strongly agree** with the wording.  In column D, please indicate any suggested revisions.  **At this stage, please consider whether the number and scope of the criteria are appropriate to address a range of EOH decision scenarios.** | | | |
| **Criteria and guidance** | | | |
| **Scoping criteria** | **Guidance for the scoping criteria** | **Is the revised scoping criterion guidance worded appropriately for scoping an EOH recommendation?** | **What additional revisions, if any, would you recommend for the wording of the guidance?** |
| **Purpose** | Specify health intents and expected benefits or outcomes. | 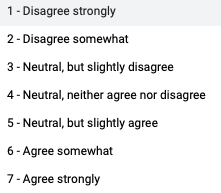 | [[FREE TEXT RESPONSE]] |
| **Perspective** | Specify the perspective that the panel will take when making recommendations: impact on a specific group (e.g., workers), or overall population/societal impact | [[see above]] | [[see above]] |
| **Target population** | Specify the intended beneficiaries of the recommendation (e.g. people affected by the environmental issue, workers, industry, society) | [[see above]] | [[see above]] |
| **Setting** | Specify level at which these recommendations will be implemented: occupational or non-occupational setting; health care or non-health care setting; regional, national, or international level | [[see above]] | [[see above]] |
| **Key coexisting conditions** | Specify the key co-exposures and susceptibility factors that may be considered when making recommendations: long working hours, age, gender, socioeconomic status, chronic illness, etc. | [[see above]] | [[see above]] |
| **Types of interventions** | Specify the intervention(s) under consideration: prevention (e.g. PPE), remediation (e.g. clean-up of contaminated areas), public education, regulation | [[see above]] | [[see above]] |
| **Key stakeholders / users** | Specify all relevant professional groups, institutions, public, etc. who are target users or beneficiaries of these guidelines and/or whose views should be sought | [[see above]] | [[see above]] |
| **Key resources** | Specify resources needed for the implementation of guidelines (i.e. need for additional human resources, equipment, infrastructure, system changes, etc.) and potential barriers to implementation. | [[see above]] | [[see above]] |
| **Key implementation issues** | Are there important barriers to implementing the intervention? Is there a communication plan to facilitate implementation? | [[see above]] | [[see above]] |
| **Existing documents** | Purposively sample existing documents/guidelines on the same or similar topic that are likely to be currently used in practice (e.g. guidelines developed by other organizations). | [[see above]] | [[see above]] |
| Any other comments | | | |
| Please add any additional thoughts or comments not covered by any of the above. | |  |  |

| **Criteria and detailed judgements** | | | | |
| --- | --- | --- | --- | --- |
| **Decision framework criteria** | **Detailed judgements** | **Should this detailed judgement be included in developing an EOH recommendation?** | **Is the revised wording for the detailed judgement appropriate for developing an EOH recommendation?** | **What additional revisions, if any, would you recommend for the wording of the detailed judgement?** |
| **Problem *Is the problem a priority?* (no, probably no, probably yes, yes, varies, don't know)** | Are the consequences of the problem serious (i.e., severe, irreversible, or important)? | 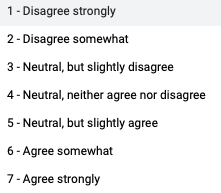 | 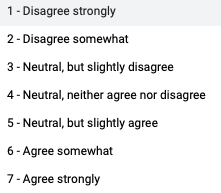 | [[FREE TEXT RESPONSE]] |
|  | Is the problem urgent, emergent, or unprecedented? | [[see above]] | [[see above]] | [[see above]] |
|  | Is the problem a recognized priority of the political system (local or national)? | [[see above]] | [[see above]] | [[see above]] |
|  | Is the problem a recognized occupational or public health concern? | [[see above]] | [[see above]] | [[see above]] |
|  | Does the problem necessitate coordination with the political or health systems of other jurisdictions? | [[see above]] | [[see above]] | [[see above]] |
|  | Is the problem a recognized priority for a local community? | [[see above]] | [[see above]] | [[see above]] |
|  | Is the community engaged in advocacy or organizing? | [[see above]] | [[see above]] | [[see above]] |
| **Desirable effects *How substantial are the desirable anticipated effects?* (trivial, small, moderate, large, varies, don't know)** | How substantial is the anticipated desirable impact (effect) of the intervention or exposure? | [[see above]] | [[see above]] | [[see above]] |
|  | What is the time span for the intervention or exposure to reach full effectiveness? | [[see above]] | [[see above]] | [[see above]] |
| **Undesirable effects *How substantial are the undesirable anticipated effects?* (large, moderate, small, trivial, varies, don't know)** | How substantial is the anticipated undesirable impact (effect) of the intervention or exposure? | [[see above]] | [[see above]] | [[see above]] |
| **Certainty of evidence *What is the overall certainty of the evidence of effects?* (very low, low, moderate, high, no included studies)** | [No detailed judgements identified for this criteria] | [[see above]] | [[see above]] | [[see above]] |
| **Values *Is there important uncertainty about or variability in how much people value the main outcomes?* (important uncertainty or variability, possibly important uncertainty or variability, probably no important uncertainty or variability, no important uncertainty or variability)** | Is there important uncertainty about how much people value the main outcomes? | [[see above]] | [[see above]] | [[see above]] |
|  | Would you expect a large range in how much people value the main outcomes? | [[see above]] | [[see above]] | [[see above]] |
|  | What is the anticipated risk tolerance among the expected beneficiary/ies of the recommendation? | [[see above]] | [[see above]] | [[see above]] |
|  | What is the anticipated risk tolerance among other stakeholder groups who are not the expected beneficiaries? | [[see above]] | [[see above]] | [[see above]] |
| **Balance of effects *Does the balance between desirable and undesirable effects favor the intervention or the comparison?* (favors the comparison, probably favors the comparison, does not favor either the intervention or the comparison, probably favors the intervention, favors the intervention, varies, don't know)** | [No detailed judgements identified for this criteria] | [[see above]] | [[see above]] | [[see above]] |
| **Resources required *How large are the resource requirements (costs)?* (large costs, moderate costs, negligible costs and savings, moderate savings, large savings, varies, don't know)** | For each type of resource, would the intervention or exposure under consideration produce additional costs or savings? | [[see above]] | [[see above]] | [[see above]] |
| **Certainty of evidence of resources required *What is the certainty of the evidence of resource requirements (costs)?* (very low, low, moderate, high, no included studies)** | Have all relevant resource types been identified? | [[see above]] | [[see above]] | [[see above]] |
|  | How certain is the evidence of anticipated resource costs and/or savings? | [[see above]] | [[see above]] | [[see above]] |
|  | Is there important variability in the cost of the resource types under consideration? | [[see above]] | [[see above]] | [[see above]] |
| **Cost effectiveness *Does the cost-effectiveness of the intervention favor the intervention or the comparison?* (favors the comparison, probably favors the comparison, does not favor either the intervention or the comparison, probably favors the intervention, favors the intervention, varies, no included studies)** | What is the certainty in the cost effectiveness analysis? | [[see above]] | [[see above]] | [[see above]] |
| **Equity *What would be the impact on health equity?* (reduced, probably reduced, probably no impact, probably increased, increased, varies, don't know)** | Are there groups or settings (e.g., susceptible populations or life stages) that may experience a different impact (effect) of the intervention or exposure due to variability in baseline conditions across the affected population? | [[see above]] | [[see above]] | [[see above]] |
|  | Are issues of equality (e.g., under-represented or under-studied groups) addressed? | [[see above]] | [[see above]] | [[see above]] |
|  | Are the impacts unreasonably disproportionate to particular groups (e.g., complex or unquantified stressors)? | [[see above]] | [[see above]] | [[see above]] |
|  | Are social justice issues addressed? (e.g., Is spirit of ‘polluter pays principle’ upheld with regard to distribution of impacts/benefits?) | [[see above]] | [[see above]] | [[see above]] |
|  | Would implementing the intervention or exposure reduce or increase inequities experienced by marginalized memebers of the affected population? | [[see above]] | [[see above]] | [[see above]] |
| **Acceptability *Is the intervention acceptable to key stakeholders?* (no, probably no, probably yes, yes, varies, don't know)** | What is the certainty in the acceptability of the intervention or exposure among the affected population? | [[see above]] | [[see above]] | [[see above]] |
|  | Is there variability in the acceptability of the intervention or exposure among beneficiaries of the recommendation and other stakeholders? | [[see above]] | [[see above]] | [[see above]] |
|  | Can intervention decisions be resolved by means of laws or regulations? | [[see above]] | [[see above]] | [[see above]] |
| **Feasibility *Is the intervention feasible to implement?* (no, probably no, probably yes, yes, varies, don't know)** | Is the intervention (option) sustainable for the relevant duration of time? | [[see above]] | [[see above]] | [[see above]] |
|  | Is there variability in the perception of feasibility of the intervention or exposure among beneficiaries of the recommendation and other stakeholders? | [[see above]] | [[see above]] | [[see above]] |
|  | Are there important barriers that are likely to limit the feasibility of implementing the intervention (option)? | [[see above]] | [[see above]] | [[see above]] |
|  | Does the presence of laws/regulations facilitate implementation of the intervention (i.e., resources and time required to pursue)? | [[see above]] | [[see above]] | [[see above]] |
|  | Does the absense of laws/regulations limit the implementability of the intervention? | [[see above]] | [[see above]] | [[see above]] |
| **Any other comments** | | | | |
| Please add any additional thoughts or comments not covered by any of the above. | | [[FREE TEXT RESPONSE]] |  |  |
